# Blocking S100A9-signaling is detrimental to the initiation of anti-tumor immunity

**DOI:** 10.3389/fimmu.2024.1479502

**Published:** 2024-10-21

**Authors:** Melike Fusun Demir, Yu-Hsien Lin, Pedro Henrique Costa Cruz, Masaki Tajima, Tasuku Honjo, Elisabeth Müller

**Affiliations:** ^1^ Department of Immunology and Genomic Medicine, Kyoto University, Kyoto, Japan; ^2^ Division of Integrated High-Order Regulatory Systems, Center for Cancer Immunotherapy and Immunobiology, Kyoto, Japan; ^3^ Tumor Immunology Group, Institute of Pathology, Oslo University Hospital, Oslo, Norway; ^4^ Therapy Prediction In Lung Cancer, Department of Cancer Genetics, Institute of Cancer Research, Oslo University Hospital, Oslo, Norway

**Keywords:** S100A9, TLR4, DAMP, monocytes, MDSC, TAMs, checkpoint inhibition, cancer immunotherapy

## Abstract

S100A9, a multifunctional protein mainly expressed by neutrophils and monocytes, poses an immunological paradox. In virus infections or sterile inflammation, it functions as an alarmin attracting innate immune cells, as well as mediating proinflammatory effects through TLR4 signaling. However, in cancer, S100A9 levels have been shown to associate with poor prognosis and lack of response to immunotherapy. Its expression by myeloid cells has been related to an immune suppressive phenotype, the so-called myeloid derived suppressor cells (MDSCs). Targeting S100A9 in cancer has therefore been proposed as a potential way to relieve myeloid-mediated immune suppression. Surprisingly, we found that blocking the extracellular TLR4 signaling from S100A9 using the inhibitor Paquinimod, resulted in increased tumor growth and a detrimental effect on anti-PD-L1 efficacy in the CT26 tumor model. This effect was caused by a reduction in the tumor immune infiltration to about half of untreated controls, and the reduction was made up of a 5-fold decrease in Ly6C^high^ monocytic cells. The suppressive Ly6G^+^ myeloid cells compartment was not reduced by Paquinimod treatment, suggesting alternative mechanisms by which S100A9 contributes to myeloid-mediated suppression. Intratumoral injection of recombinant S100A9 early after mice inoculation with CT26 cells had an anti-tumor effect. These findings indicate an important yet understudied role of S100A9 as an alarmin and immune stimulatory signal in cancer settings, and highlight the potential to exploit such signals to promote beneficial anti-tumor responses.

## 1 Introduction

The alarmin S100A9 has been described to induce an immune suppressive phenotype in myeloid cells during cancer, which run contrary to its role as a proinflammatory signal in infections or sterile inflammation. S100A9 and other self-derived immunomodulatory compounds released upon tissue damage or stress, work to “sound the alarm” and initiate subsequent immune responses (1, 2). S100A9 proteins in the form of homodimers or heterodimers with S100A8, are expressed at high levels in neutrophils and monocytes, and are also released upon activation or degranulation (3, 4). S100A9 homodimer and S100A8/A9 heterodimer are reported to activate TLR4, resulting in activation and attraction of additional immune cells (5). S100A8 and S100A9 have been shown to mediate both beneficial defense against pathogens and pathological inflammation in autoimmune diseases, demonstrating their proinflammatory potential (6). In cancer however, S100A9 has received most attention for its role in tumor-promoting inflammation (7), and in the development and recruitment of myeloid derived suppressor cells (MDSCs), which limit beneficial anti-tumor immunity (8, 9).

In cancers, abnormal activation of myeloid cells has been related to the induction of an immune suppressive phenotype, where these cells can inhibit T cell infiltration (10). Suppressive myeloid cells have been shown to be a major obstacle for T cells to entry into tumors, which is critically impacting the efficacy of cancer immunotherapy (11). Research from both the Gabrilovich lab and Ostrand-Rosenberg lab has identified S100A8 and S100A9 as factors which contribute to differentiating immature myeloid cells to become MDSCs and the accumulation of these cells during cancer progression (8, 9). Similarly, NF- κB activation following TLR stimulation, IL-1β and IL-6, have been shown to promote an immune-suppressive phenotype in these myeloid cells, despite being typically recognized as pro-inflammatory signals (12, 13). It is unclear whether these signals per se induce the expression of regulatory mediators, such as IL-10 or prostaglandin E2 (PGE2) (14), or whether the activation of myeloid cells at a specific time during their differentiation causes the suppressive phenotype (15). Chronic or smoldering inflammation is recognized as a hallmark of cancers (16), and S100 proteins have been suggested to serve as potential biomarkers of systemic or local detrimental inflammation (17, 18). Increased levels of S100A9 were found in plasma in many cancer patients, and some studies have showed correlations between S100A9 levels and tumor progression (19) or resistance to checkpoint inhibition (18).

Blocking the effect of S100A8 and/or S100A9 in cancer-related inflammation could potentially relieve myeloid-mediated immune suppression and synergize with other cancer immunotherapies. Previous work by Fredrik Ivars and Thomas Leanderson et. al, on a group of quinoline-3-carboxamide drugs, known for reducing inflammation and symptoms of autoimmune disease, led to the discovery of binding by S100A9 to TLR4 as a main target of these drugs (20). Tasquinimod, one of such drugs, was found to reduce MDSCs and have some anti-tumor effect in mouse models of cancer when combined with vaccine or tumor-targeted superantigen (21). Paquinimod, a structurally similar compound to Tasquinimod, has been studied mainly in autoimmune context, where was shown to reduce pathology in lupus erythematosus (22), pulmonary fibrosis (23), neutrophilic asthma (24), models of diabetes (25) and inflammatory bowel disease (26). More recently, Paquinimod was also shown to have strong immune-modulatory effects in sepsis (27), and severe COVID-19 infection (28).

In this study, we asked if Paquinimod, for which direct binding to S100A9 protein and TLR4 receptor inhibition activity was first published (20), could impact on suppressive myeloid cells and achieve anti-cancer efficacy. We tested the Paquinimod effect alone or in combination with the checkpoint inhibitor anti-PD-L1. Thus, we injected Paquinimod along with tumor cells in mice and analyzed the frequency and phenotype of myeloid cells, at both tumor sites and spleens at various times post-tumor challenge. Contrary to our expectations, Paquinimod treatment showed a pro-tumor effect in two different tumor models, with mice injected with the drug having significantly larger tumors compared to untreated mice.

Although immune cell populations in spleen were unaltered by Paquinimod treatment, we observed an overall reduction in immune cell infiltration (CD45^+^ cells) into the tumors, to less than half compared to untreated controls. Ly6C^high^ CD11b^+^ myeloid cells at tumor sites were strongly reduced upon Paquinimod treatment. These cells, likely to constitute early inflammatory monocytes, made up about 25% of total cells in untreated tumors, but were reduced to about 5% of total cells in Paquinimod treated tumors. The frequencies of both CD4^+^ T cells and CD8^+^ T cells were also significantly reduced by Paquinimod, indicating that treated tumors turned less immunologically “hot”. To investigate if we could reverse the Paquinimod effect and improve the anti-tumor immune responses, we tested intratumoral injection of recombinant S100A9 protein. Increased levels of S100A9 in the tumor resulted in significantly reduced tumor growth. *In vitro*, Paquinimod reduced the chemotactic response of Ly6C^high^ myeloid cells to recombinant S100A9 and CT26 tumor supernatant, strongly suggesting that Paquinimod might promote tumor growth through blocking chemotactic signals from tumor, through binding to S100A9.

These findings indicate that extracellular S100A9-TLR4 signaling function as a beneficial chemotactic signal for proinflammatory Ly6C^high^ myeloid cells into tumors, driving the infiltration of subsequent immune cells during early stages of tumor development. Future studies will be needed to address the role of S100A9-TLR4 signaling at later stages of tumor development in order to understand how this signaling pathway works in chronic settings. Nonetheless, our study underlines the multifaceted roles played by the alarmin S100A9 in tumor immunity and myeloid cell regulation, with both beneficial and detrimental effects, and the need to identify alternative inhibitors or pathways to abolish the generation of suppressive myeloid cells in cancer.

## 2 Methods

### 2.1 Mice

Wild-type BALB/c and C57BL/6N mice, aged 8 to 16 weeks, were obtained from Japan SLC. All mice were used in accordance with protocols approved by the respective institutional review board (IRB). They were housed under specific pathogen-free (SPF) conditions, with ad-libitum water and food supply to maintain their health and well-being.

### 2.2 Cell lines

CT26 and LLC cell lines were cultured in RPMI-1640 medium (catalog # 189-02025, FUJIFILM Wako Pure Chemical Co., Ltd. Wako) with 10% (v/v) FBS (Lot # 2166446, Gibco) and 1% (v/v) Penicillin/Streptomycin mixed solution (catalog # 26253-84, Nacalai Tesque).

### 2.3 *In vivo* tumor challenge experiments

CT26 (1×10^6^) or LLC (5×10^5^) cells were injected intradermally (i.d.) into the right flank of BALB/c or C57BL/6N mice respectively on day 0. Mice were treated with Paquinimod (catalog # SML2883-25MG, Sigma Aldrich) dissolved in DMSO (catalog # 031-24051, FUJIFILM Wako Pure Chemical Co., Ltd. Wako) and diluted to 1mg/ml in PBS (catalog #14249-24, Nacalai Tesque) by intraperitonal (i.p.) injection daily, from day 0 to day 11. Control mice were injected with solvent solution (DMSO diluted in PBS). For CPI treatment, mice were injected i.p. with anti-PD-L1 monoclonal antibody (clone 1-111A, produced in-house, 50 µg/mouse) every 3 days, starting day 6 after tumor inoculation. Treatment with recombinant mouse S100A9 protein, CF (R&D, catalog # 2065-S9) (2 µg/mouse in 20 µl in PBS) was administered intratumorally on days 7 and 11. Control mice received an intratumoral injection of an equal volume (20 µL) of PBS (Nacalai Tesque). Tumor volumes were measured every other day using electronic calipers, and the tumor volume was calculated using the formula: tumor volume = π × (length × breadth × height)/6. Mice were sacrificed either on day 12 for early analysis of tumor-infiltrating immune cells in tumor, or on day 19-21 for growth curve analysis, total tumor and spleen weight and late analysis of immune cells in spleen and tumor.

### 2.4 Sample preparation for ex vivo analysis

Mice were euthanized and tumors and spleens harvested into tubes containing RPMI-1640 medium. Tumor samples processed by first mincing into 1- to 2-mm pieces using scissors and then digestion using collagenase type II (catalog # CLS2, Worthington Biochemical Corporation) (100 units/mL) and DNase I (catalog # 11284932001, Roche) (20 µg/mL) on a gentleMACS Dissociator (catalog # 130-093-235, Miltenyi Biotec). The digested tumor tissue was passed through a 100 μm filter to generate a single cell suspension, washed and resuspended in RPMI-1640 with 10% FBS. Spleens were homogenized by mashing through a 100 µm filter and subsequently treated with ammonium-chloride-potassium (ACK) (catalog # A1049201, Thermofisher Gibco™) lysing buffer for 5 minutes to remove red blood cells. Spleen cell suspensions were then washed and resuspended in RPMI with 10% FBS.

### 2.5 Flow cytometry

Single cell suspensions obtained from harvested organs or primary cell cultures were seeded on v bottom plates, washed in PBS and stained for 10 min at room temperature using the Zombie Aqua™ Fixable Viability Kit (catalog # 423102, BioLegend) to evaluate cell viability. Subsequently, cells were washed with a buffer solution containing PBS, 2% FBS, and 2 mM EDTA (catalog #06894-85, Nacalai Tesque), and treated with Trustain FcX (BioLegend, catalog #101320) for 10 minutes at 4°C, to minimize non-specific binding by blocking Fc receptors. Finally, cells were stained with a master mix of fluorescent antibodies for 30 minutes at 4°C, washed and resuspended in flow buffer. FMO controls were used to define positive and negative events. Samples were analyzed on the SONY ID7000 spectral analyzer and data processing was performed using FlowJo software v10.10 (BD Biosciences). Quantitative analysis of the flow cytometry data is detailed in the “Statistical analysis” section below.

Cells were stained with the following antibodies: Anti-FoxP3 (catalog #11-5773-82, clone PJK-169, Invitrogen), Anti-I-A/I-E(catalog #107606, clone M5/114.152, BioLegend), Anti-CD11b (catalog #101226, clone M1/70, BioLegend), Anti-CD45 (catalog #564279, clone 30-F11, BioLegend), Anti-CD86 (catalog #564199, clone 6L1, BioLegend), Anti-CD8a (catalog #50-1886-U100, clone 2.43, Tonbo Biosciences), Anti-F4/80 (catalog #123110, clone BM8, BioLegend), Anti-CD80 (catalog #104708, clone 16-10A1, BioLegend), Anti-CD4 (catalog #100536, clone RM4-5, BioLegend), Anti-Arg-1 (catalog #17-3697-82, clone A1exF5, Invitrogen), Anti-Ly6G (catalog #127622, clone 1A8 BioLegend), Anti-Ly6C (catalog #560593, clone AL-21, BD Pharmingen), Anti-CD274 (catalog #124336, clone 10F.962, BioLegend), Anti-CD279 (catalog #135218, clone 29F.1A12, BioLegend).

### 2.6 Magnetic-activated cell sorting

Ly6G^+^ cells from the spleens were isolated from spleen single-cell suspensions by employing the Anti-Ly-6G MicroBeads UltraPure kit (catalog #130-120-337, Miltenyi Biotec) following the manufacturer’s recommended protocols. Spleens from naive, control, and Paquinimod (Sigma-Aldrich) treated mice were harvested and pooled based on treatment groups. The spleens were mechanically dissociated by pressing them through a 70 µm cell strainer using a syringe plunger to obtain a single-cell suspension. The cell suspension was centrifuged at 400×g for 5 minutes, and the pellet was resuspended in ACK (Ammonium-Chloride-Potassium) lysis buffer (Gibco) to lyse red blood cells. After another centrifugation at 400×g for 5 minutes, the cells were resuspended in a buffer containing phosphate-buffered saline (PBS) (Nacalai Tesque), 0.5% bovine serum albumin (BSA) (catalog # A8531, Sigma-Aldrich), and 2 mM EDTA (Nacalai Tesque), The cells were labeled with Anti-Ly-6G MicroBeads UltraPure (Miltenyi Biotec) by adding 10 µL of MicroBeads per 10^7^ cells and incubating at 4°C for 10 minutes. After washing to remove unbound MicroBeads, the cell suspension was applied to a MACS^®^ LS Column (catalog #130-042-401, Miltenyi Biotec) placed in the magnetic field of a MACS Separator (catalog # 130-042-303, Miltenyi Biotec). The labeled Ly6G^+^ cells were retained in the column, while unlabeled cells passed through. The column was removed from the magnetic field, and the retained Ly6G^+^ cells were eluted.

### 2.7 T cell suppression assay

Effector Ly6G^+^ cells were generated from single-cell suspensions of spleens from both treated and naïve mice using Ly6G^+^ beads sorting, as described above. A portion of naïve splenocytes from non-tumor bearing mice were kept unsorted for use as target cells. Effector Ly6G^+^ cells of interest were seeded in triplicates in varying numbers in U-bottom 96-well plates (catalog # 353077, Corning Incorporated) in RPMI-1640 medium (Wako) supplemented with 10% (v/v) heat-inactivated fetal bovine serum (FBS) (Gibco) and 1% (v/v) penicillin-streptomycin mixed solution (Nacalai Tesque). Target cells, consisting of total splenocytes harvested from naïve mice, were stained with the CellTrace-CSFE Cell Proliferation kit (catalog # C34554, Invitrogen) and added at 200.000 cells/well density to the effector cells, yielding different ratios of effector to target cells. T cells in the splenocyte target population were stimulated by adding anti-CD3 and anti-CD28 monoclonal antibody-coated Dynabeads (catalog # 11453D, Thermo Fisher Scientific, Gibco). The co-cultures were incubated for 4 days at 37°C in 5% CO_2_. Subsequently, the cells were harvested, stained for CD11b (catalog # 101226, clone M1/70, BioLegend), CD4 (catalog # 100536, clone RM4-5, BioLegend) and CD8 Anti-CD8a (catalog # 50-1886-U100, clone 2.43, Tonbo Biosciences) and analyzed using the SONY ID7000 spectral cell analyzer. Proliferation was determined by measuring the decrease in CellTrace-CFSE fluorescence intensity in CD4^+^ and CD8^+^ T cells. Splenocytes stimulated with anti-CD3 and anti-CD28 monoclonal antibody-coated Dynabeads alone was used as the control and were considered 100% proliferated.

### 2.8 Migration assay

Ly6C^high^ cells from bone marrow cells were obtained from the femur and tibia of naïve C57BL/6 mice by flushing the bones with PBS containing 2% FBS and 2 mM EDTA. After lysing erythrocytes, the remaining cells were washed and resuspended in PBS with 2% FBS and 2 mM EDTA for fluorescence-activated cell sorting (FACS). Bone marrow cells were stained with Zombie Aqua (catalog # 423102, BioLegend) for identification of live cells, followed by blocking Fc receptors with anti-CD16/32 (catalog #101320, BioLegend) antibodies. The following antibodies were used for subsequent staining: Anti-CD11b APC-Cy7 (catalog # 101226, clone M1/70, BioLegend), Anti-Ly6G APC (catalog # 127614 clone 1A8, BioLegend), Anti-Ly6C PE-Cy7 (catalog #560593, clone AL-21, BD Pharmingen), and Anti-CD45 FITC (catalog # 103108, clone 30-F11, BioLegend). The Ly6C^high^ target cell population to be used in the migration assay, identified as Zombie^–^CD45^+^CD11b^+^Ly6G^–^Ly6C^high^ cells, was sorted using a BD FACSMelody^™^ sorter (BD Biosciences). The purity of the sorted cells was confirmed, and cells were resuspended in RPMI-1640 containing 10% FBS (Gibco) and 1% pen/strep (Nacalai Tesque) for use in the Boyden chamber migration assay. Gating strategy for sorting and results from the purity assessment is shown in **Supplementary Figures 8** and **9**.

The Boyden chamber assay was performed using upper and lower compartments separated by a porous membrane with an 8 μm pore size (catalog # 3422, Corning Incorporated). FACS- sorted Ly6C^high^ bone marrow cells were seeded in upper chamber (insert). To test the effect of the inhibitor, cells in the lower chamber were treated with or without various doses of the inhibitor under these conditions: RPMI-1640 (Wako) medium alone (negative control), RPMI-1640 (Wako) medium with the chemoattractant complement C5a (catalog # 2150-C5-025/CF, R&D) (1 µg/mL, positive control), RPMI-1640 medium with recombinant mouse S100A9 protein (R&D), CT26 tumor cell line supernatant, CT26 supernatant with recombinant mouse S100A9 protein (R&D). The CT26 supernatant was collected from CT26 cells cultured to confluence in T75 flasks. After reaching confluence, the supernatant was harvested, centrifuged at 400 g for 5 minutes to remove residual cells, and filtered through a 28 mm 0.45 µm SFCA membrane syringe filter (catalog # 431220, Corning Incorporated) to ensure purity. The inhibitor was added to the lower chamber at various doses, to assess its effect on migration under these conditions.

After 24 hours of incubation at 37°C in 5% CO2, migrated cells in the lower chamber were stained with NucBlue™ Live ReadyProbes™ Reagent-Hoechst 33342, (catalog # R37605, Invitrogen) as per the manufacturer’s instructions. Briefly, cells were stained with 1 drop/2 mL NucBlue™ Live ReadyProbes™ Reagent-Hoechst 33342 and incubated for 15 minutes at 37°C and 5% CO2. Images of the Transwell lower chamber bottom were taken with a 10x objective using a Keyence BZ-X800 All-in-One Fluorescence microscope (Keyence Corporation). The number of migrated cells stained with NucBlue™ Live ReadyProbes™ Reagent-Hoechst 33342 was counted using Keyence BZ-800 image analysis software Hybrid Cells count module and Macro cell count options (Keyence Corporation).

### 2.9 Statistical analysis

Statistical analysis was performed using Prism v9.3 (Graphpad). Treatment groups were compared using the two-sided unpaired *t* test (for two groups), or one-way or two-way ANOVA (for data with one or two independent variables, respectively) with Tukey’s multiple comparisons test (for more than two groups). No technical replicates were used to derive statistics in this study. All statistical analyses have been performed using 3 or more biological replicates. For all experiments, the alpha level was set to 0.05. All *p* values are displayed, with *, ** and *** indicating *p* < 0.05, *p* < 0.01 and *p* < 0.001, respectively.

## 3 Results

### 3.1 Responsiveness of CT26 to anti-PD-L1 treatment inversely correlates with induction of immune suppressive myeloid cells

**Figure 1:**
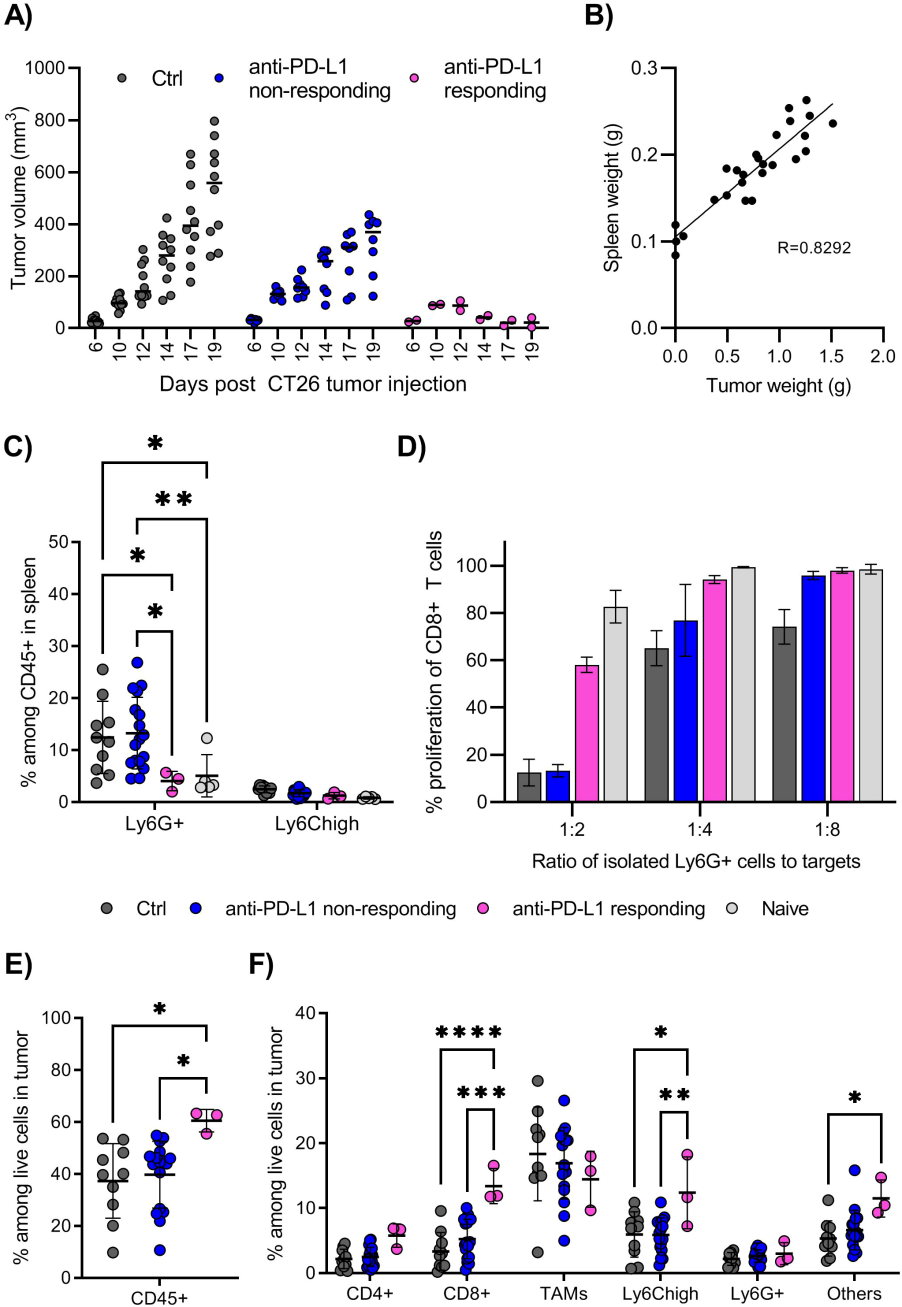
The CT26 tumor model is partially responsive to CPI therapy. **(A)** Growth of CT26 tumors in individual mice, grouped depending on treatment and response. BALB/c mice were injected with CT26 tumor cells intradermally and treated with anti-PD-L1 monoclonal antibody every 3 days starting at day 6, or left untreated. Treated mice in which the tumors showed reduction in size in minimum two repeated measurements, were denoted “responders”, and the remaining treated mice denoted “non-responders”. Tumor volume is calculated as l*w*h*3,14/6. N=10 mice for untreated control and anti-PD-L1 treated mice in total. Graph shows representative experiment from 4 repeats. **(B)** Correlation between spleen weight (g) and tumor weight (g) in untreated mice at day 18-20 post tumor injection. N=26, pooled results from 3 experiments. **(C)** Frequency of Ly6G^+^ and Ly6C^high^ myeloid cells in spleen, harvested and processed for flow cytometry analysis on day 18-20 post tumor injection. Gating strategy for immune cell subsets in spleen is shown in **Supplementary Figure 1**. N=10 mice for untreated control, 17 for CPI non-responding, 3 for CPI responding and 5 for naïve, non-tumor bearing mice. Graph shows representative experiment from 3 repeats. **(D)** T cell suppression assay using Ly6G^+^ cells isolated from spleen as effector cells and splenocytes from naïve mice as target cells. Overview of the experimental setup is shown in **Supplementary Figure 2**. Spleens from 10, 8, 2 or 5 mice (Ctrl, CPI non-responding, CPI responding, naïve) were harvested day 18 post tumor injection and pooled prior to Ly6G isolation. Graphs shows representative experiment from 3 repeats with error bars showing standard deviation from technical repeats, N=3. **(E)** Frequency of total immune cells in tumors, identified as CD45^+^ among live cells by flow cytometry. **(F)** Frequency of immune cell subsets among total live cells in tumors. Gating strategy for immune cell subsets in tumor is shown in **Supplementary Figure 3**. **(E, F)** Graph shows representative experiment from 3 repeats, N=10, 17 and 3 for control, CPI non-responding and CPI responding. Statistically signi!cant differences between treatment groups are denoted *, **, *** or **** depending on p-values as described in methods section.

During initial investigations, we searched for correlations between the responsiveness to anti-PD-L1 responsiveness and immune cell infiltration in multiple tumor models. The mice injected with CT26 colon carcinoma cell line showed typically two patterns of tumor growth: a small number of mice (2 out of 10 mice) responded well to anti-PD-L1 treatment and rejected the tumors, while the majority of mice showed only a partial response to the treatment, with tumors progressing, although at reduced speed (**Figure 1A**). We found that the tumor burden tightly correlated with splenomegaly (**Figure 1B**). The main cellular subset associated with splenomegaly was that of Ly6G^+^ granulocyte-like cells, which were significantly increased in tumor-bearing mice compared with the naïve mice, or mice responding to anti-PD-L1 treatment and rejecting the tumors (**Figure 1C**). Other immune cell subsets evaluated, like CD8^+^ T cells, CD4^+^ T cells or macrophages were not altered (data not shown, gating strategy shown in **Supplementary Figure 1**). The expanded splenic Ly6G^+^ cells from tumor-bearing mice showed typical functional phenotype of MDSCs, as they effectively inhibited the proliferation of T cells ex vivo (**Figure 1D**). Such immunosuppressive phenotype was not observed with Ly6G^+^ cells from naïve, non-tumor bearing mice (**Figure 1D**, suppression assay outline shown in **Supplementary Figure 2**). In anti-PD-L1 non-responding mice, both the frequency and the suppressive function of Ly6G^+^ cells were similar to that of untreated tumor-bearing mice (**Figure 1C**). In contrast, anti-PD-L1 responding mice, lacked both the expansion of Ly6G^+^ cells as well as the suppressive function of these cells, which were similar to those in naïve mice (**Figures 1C, D**).

The frequencies of immune cells infiltrating the CT26 tumors were quite high, with CD45^+^ cells making up about 40% of live cells, with further significant increase to 60% in mice responsive to anti-PD-L1 therapy (**Figure 1E**). A large proportion of tumor-infiltrating immune cells were myeloid cells, mainly F4/80^+^ tumor associated macrophages (TAMs) and monocytic (Ly6C^high^), with smaller populations of CD4^+^ T cells, CD8^+^ T cells and granulocytic (Ly6G^+^) myeloid cells (**Figure 1F**, gating strategy shown in **Supplementary Figure 3**). Anti-PD-L1-responsive mice showed a significantly higher frequency of CD8^+^ T cells and Ly6C^high^ myeloid cells infiltrating the tumor, indicating that these cells contribute to a successful anti-tumor immune response, in addition to the absence of systemic Ly6G^+^ suppressive cells. Thus, the CT26 appears to be a good model to test drugs aiming of inhibiting the generation and function of suppressive myeloid cells and improve efficacy of checkpoint inhibitor (CPI) therapies.

### 3.2 Paquinimod treatment promotes tumor growth and resistance to anti-PD-L1 therapy

Previous studies, utilizing genetic knockdown, monoclonal antibodies and pharmaceutical inhibitors, have identified the protein S100A9 or the protein complex S100A8/A9 as playing a major role in the generation of MDSCs as well as suppressive TAMs (8, 9, 29). S100A9 either as homodimers or heterodimers with S100A8, were found binding and signaling through TLR4, in addition to RAGE and other receptors (2, 30). The interaction between S100A9 and TLR4, and possibly also with the receptor RAGE, can be blocked using the inhibitor Paquinimod, a quinoline-3-carboxamide derivative. Due to its previous testing in a clinical setting, safe clinical profile and blocking of both human and mouse S100A9 signaling through TLR4, we decided to investigate this drug. Specifically, we tested whether Paquinimod could target the detrimental differentiation of myeloid cells into MDSCs and/or reduce the systemic accumulation of these suppressive myeloid cells, leading to the suppression of anti-tumor immunity and CPI resistance.

**Figure 2:**
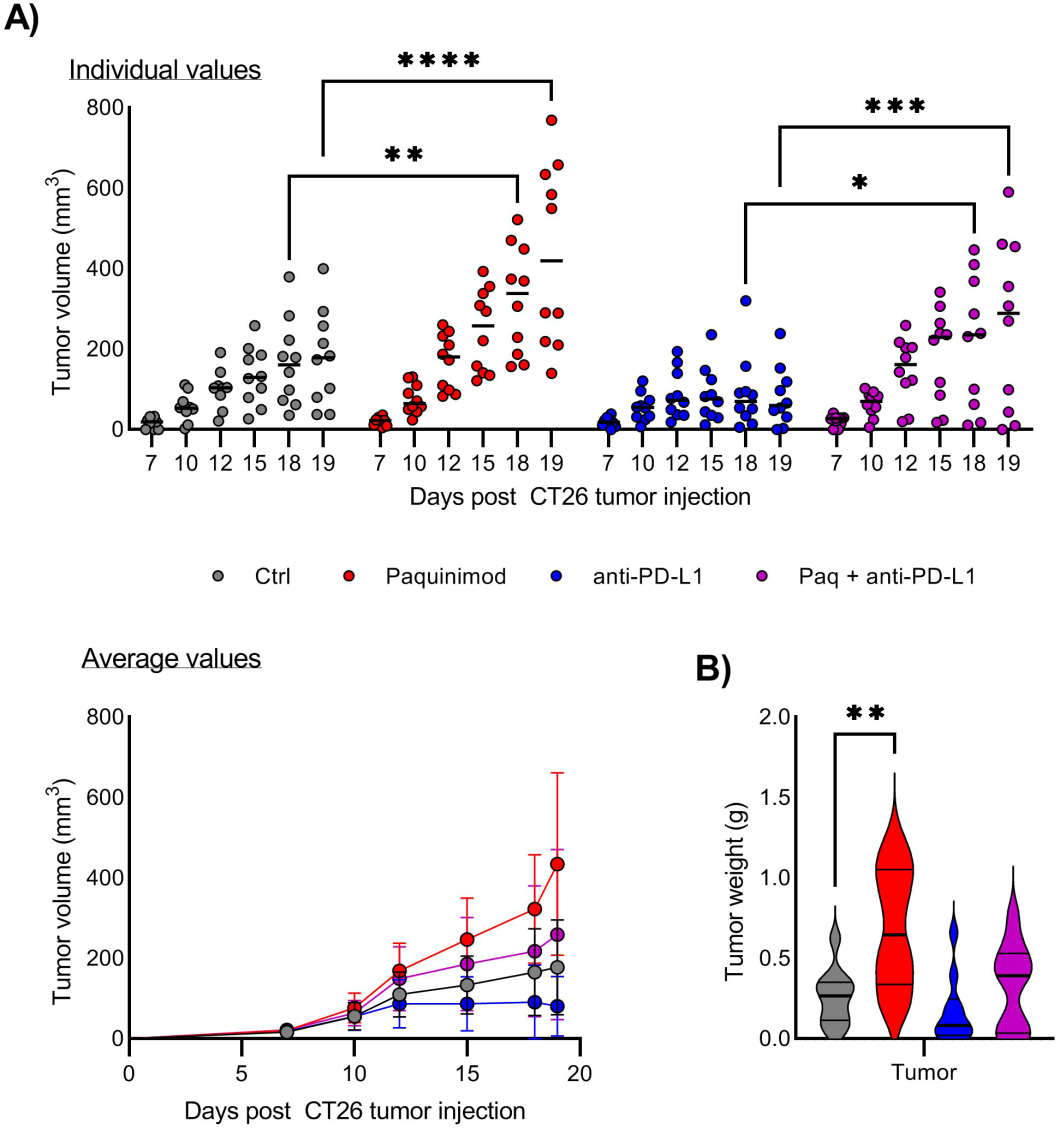
Effect of Paquinimod treatment on CT26 tumor growth. **(A)** Growth of CT26 tumors depending on treatment and response. Top panel shows tumor growth of individual mice, and lower panel tumor growth as average ^+^/- SD. BALB/c mice were injected with CT26 tumor cells intradermally and treated with Paquinimod day 0-11 and/or anti-PD-L1 monoclonal antibody every 3 days starting at day 6 or left untreated. Experimental setup is shown in **Supplementary Figure 4**. Tumors were measured every 2-3 days and tumor volume is calculated as l*w*h*3,14/6. N=10. Graph shows representative experiment from 3 repeats. **(B)** Tumor weight at harvest day 19. Statistically significant differences between treatment groups are denoted *, **, *** or **** depending on p-values as described in methods section.

The mice were injected with Paquinimod intraperitoneally, from day 0 to day 12 after CT26 tumor inoculation (**Supplementary Figure 4**). Contrary to our expectations, Paquinimod treatment resulted in increased CT26 tumor growth and also eradicated the responsiveness of CT26 to anti-PD-L1 (**Figure 2**). There were individual variations within the treatment groups, but Paquinimod injection alone or in combination and anti-PD-L1, resulted in even larger variations (maximal and minimal) tumor volume within the respective groups (**Figure 2A**). In mice injected with Paquinimod, we observed a significant increase in tumor volume from day 15 (**Figure 2A**) and a significant doubling in average tumor weight at day 19 (**Figure 2B**). Thus, combining Paquinimod with CPI resulted in increased tumor growth in non-responding mice and abrogation of the overall reduction in tumor growth compared with control mice. The protumor effect of Paquinimod was reproduced in the LLC tumor model (**Supplementary Figure 5**). Paquinimod injection at later timepoints showed no pro or anti-tumor effect, and neither affected the growth of CT26 tumor cells *in vitro* (data not shown). Thus, it seems that there is a critical time-window during early tumor growth, for a beneficial effect of S100A9 on anti-tumor control, which is blocked by Paquinimod. These findings suggest that extracellular S100A9 signaling restricts tumor growth indirectly and contribute to CPI response, through early immune-mediated control of tumor growth.

### 3.3 Paquinimod does not affect generation of suppressive Ly6G^+^ cells *in vivo*

**Figure 3:**
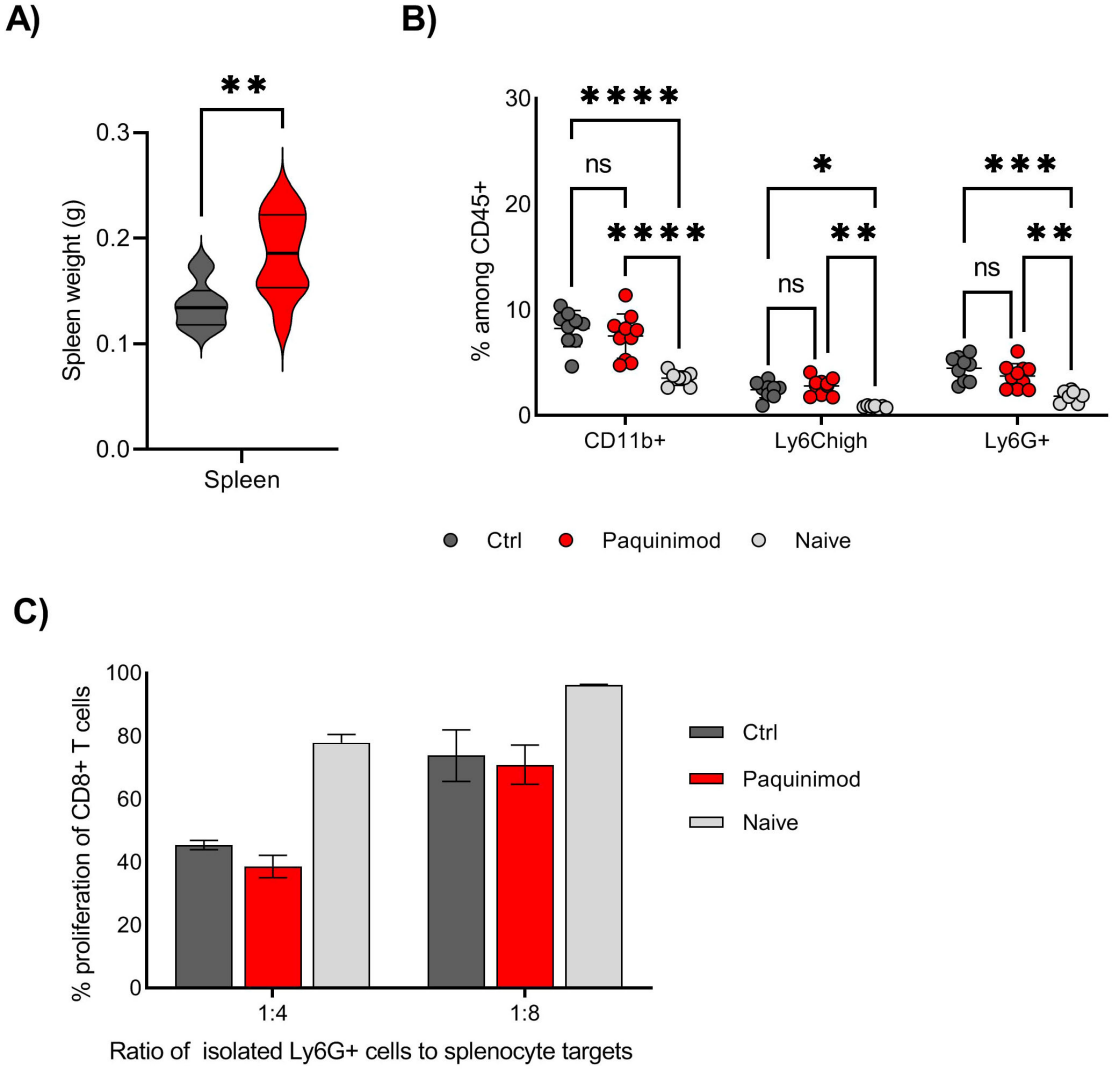
Effect of Paquinimod treatment on spleen and myeloid cells. **(A)** Average spleen weight at harvest day 19. BALB/c mice were injected with CT26 tumor cells intradermally and treated with Paquinimod day 0-11 or left untreated. N=10. Graph shows representative experiment from 3 repeats. **(B)** Frequency of total CD11b^+^ cells, Ly6G^+^ and Ly6C^high^ myeloid cells in individual spleens, harvested at day 19. N=10, 10 and 7. Graph shows representative experiment from 3 repeats. Statistically significant differences between treatment groups are denoted *, **, *** or **** depending on p-values as described in methods section. **(C)** T cell suppression assay using Ly6G^+^ cells isolated from spleen as effector cells and splenocytes from naïve, non-tumor bearing mice as target cells. Spleens from 10, 10 and 7 mice (Ctrl, Paquinimod treated and naïve) were harvested day 19 post tumor injection and pooled prior to Ly6G isolation. Graphs shows representative experiment from 3 repeats with error bars showing standard deviation from technical repeats, N=3. ns, not significant.

Following the results from the tumor challenge experiments, we were curious to know whether the induction of suppressive myeloid cells, i.e. spleen Ly6G^+^ myeloid population, was affected by Paquinimod treatment. Based on a previous study reporting the role of S100A8/A9 in sustaining the accumulation of MDSC in all lymphoid tissues, we expected that Paquinimod would result in a systemic reduction of the tumor-induced expansion of myeloid cells (8). We found, however, that Paquinimod injection alone did not impact the expansion of myeloid cells, including Ly6G^+^ cells in the spleen, and the splenomegaly was not reduced but rather exacerbated by Paquinimod (**Figures 3A, B**).

T cell proliferation assays using Ly6G^+^ cells isolated from spleens of control or Paquinimod-treated tumor bearing mice, as well as naïve, non-tumor bearing mice, revealed that Paquinimod did not change the function of spleen Ly6G^+^ cells, which suppressed T cells to the same degree as Ly6G^+^ from tumor-bearing control mice (**Figure 3C**). We conclude that Paquinimod treatment does not provide any relief from myeloid immune suppression and that the generation of both suppressive Ly6G^+^ and BM-MDSCs likely do not require extracellular S100A9 signaling through TLR4.

### 3.4 Paquinimod treatment reduces immune cell infiltration into tumor

To explore as to why Paquinimod treatment caused increased tumor growth, we harvested tumor tissue on day 12, and analyzed the infiltrating immune cells by flow cytometry. It is worth noting that at this timepoint, the tumor size was relatively similar between control and treated groups. This is important, as we have observed variations in immune cell infiltration in untreated tumors that correlate with tumor size (data not shown). By analyzing at day 12, an early timepoint, we expect most variations in tumor-infiltrating cells to be caused by the treatment and not by the differences induced by variations in tumor mass and their potential to influence local and systemic immune system.

**Figure 4:**
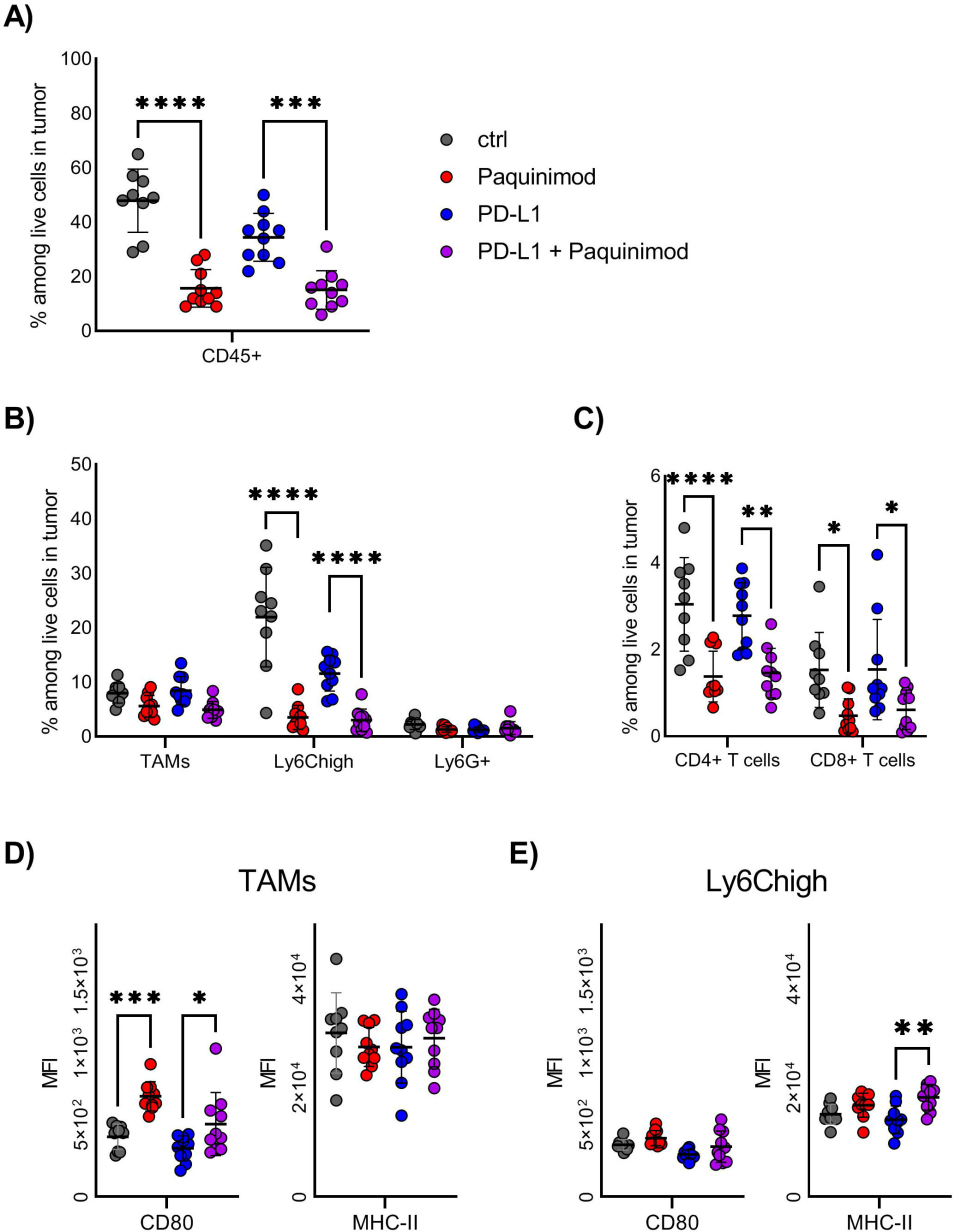
Effect of Paquinimod treatment on tumor-infiltrating immune cells. **(A–E)** BALB/c mice were injected with CT26 tumor cells intradermally and treated with Paquinimod day 0-11 or left untreated. Tumors were harvested at day 12. N=9 mice for untreated control and 10 for Paquinimod, CPI and Paquinimod ^+^ CPI. Graph shows representative experiment from 3 repeats. **(A)** Frequency of total immune cells in tumors, identified as CD45^+^ among live cells by flow cytometry. **(B)** Frequency of myeloid immune cell subsets among total live cells in tumors. **(C)** Frequency of T cell subsets among total live cells in tumors. **(D)** Mean fluorescence intensity (MFI) of CD80 and MHC-II on TAMs from tumors. **(E)** Mean fluorescence intensity (MFI) of CD80 and MHC-II on Ly6C^high^ cells from tumors. Statistically significant differences between treatment groups are denoted *, **, *** or **** depending on p-values as described in methods section.

We found that the frequency of tumor-infiltrating immune cells among live cells was reduced by more than half in Paquinimod-treated groups, compared with non-treated or anti-PD-L1 only control groups (**Figure 4A**). This result indicates that the pro-tumor effect of Paquinimod treatment is caused by lower immune cell infiltration, causing a less effective anti-tumor immune response. The composition of immune infiltrating cells was also changed by Paquinimod treatment. The frequency of Ly6C^high^ myeloid cells, the most prevalent immune cell among live cells in tumor at this early timepoint, was strongly reduced in both Paquinimod and anti-PD-L1 and Paquinimod groups compared with untreated and PD-L1 groups, respectively (**Figure 4B**). Also, there were smaller, albeit significant reductions in the frequency of both CD4^+^ T cells and CD8^+^ T cells (**Figure 4C**).

Both TAMs and Ly6C^high^ express high levels of TLR4, one important receptor engaged by extracellular S100A9 (2). We investigated if these cell subsets were altered in their activational state, by means of functional marker expression. We found no significant differences in the expression of the immune suppressive markers, like arginase (Arg)1 and CD206 (data not shown). For the immune activating markers, like CD80 and MHC-II, we found an increase in CD80 expression for TAMs and MHC-II expression for Ly6C^high^ after Paquinimod treatment (**Figures 4D, E**), but this did not reach a statistic significance. We conclude that, the strongest effect of Paquinimod treatment in this experimental setup is considerable reduction of Ly6C^high^ monocytic cells, associated with an overall reduction in immune cell infiltration.

### 3.5 Intratumoral injection of recombinant S100A9 reduces tumor growth

The findings above suggest that S100A9 signaling would be beneficial for the initial anti-cancer immune responses. We tested this hypothesis, by directly injecting recombinant S100A9 into the tumors. We chose to do intratumoral injection of the protein at an early therapeutic window, that is, at day 7 after tumor inoculation, when the tumor size is big enough to control the injection site, and then, at day 11, just before the rapid tumor cells expansion, which typically start at day 12.

**Figure 5:**
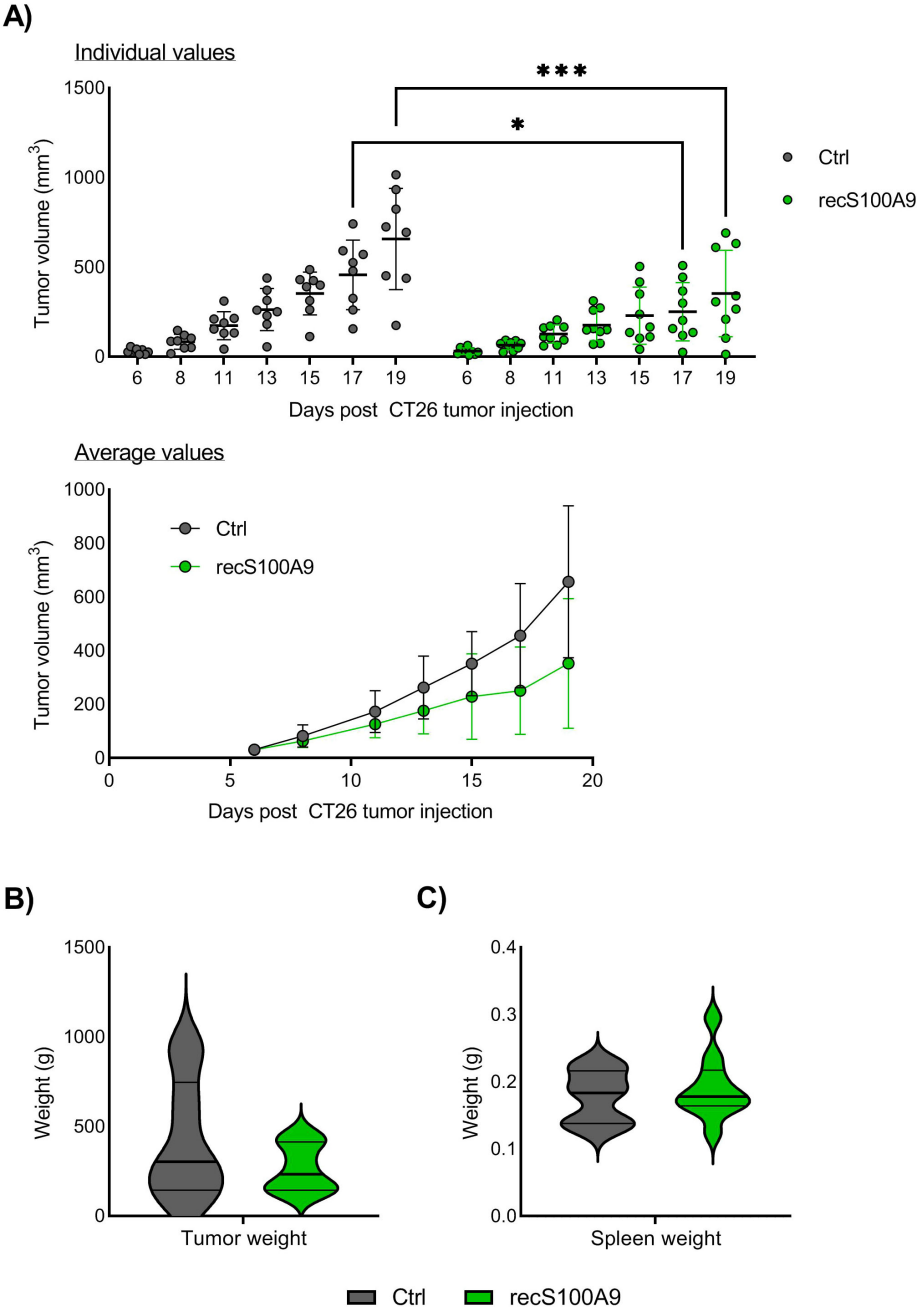
Effect of intratumoral injection of recombinant S100A9 on CT26 tumor growth. **(A)** Growth of CT26 tumors depending on treatment. Top panel shows tumor growth of individual mice, and lower panel tumor growth as average ^+^/- SD. BALB/c mice were injected with CT26 tumor cells intradermally and treated with recombinant S100A9 (recS100A9) protein on day 7 and day 11 by intratumoral injection left untreated. Experimental setup is shown in **Supplementary Figure 6**. Tumors were measured every 2-3 days and tumor volume is calculated as l*w*h*3,14/6. N=8 (untreated control) and 9 (recS100A9). Graph shows representative experiment from 3 repeats. **(B)** Tumor weight at harvest day 20. **(C)** Tumor weight at harvest day 20. Statistically significant differences between treatment groups are denoted * or *** depending on p-values as described in methods section.

We found that recombinant S100A9 injection significantly reduced CT26 tumor growth from day 17, compared with untreated controls (**Figure 5A**). When examining individual mice, there was large variation within the groups, with typically only 1 responder among 9-10 mice that completely rejected the tumor and with the remaining mice clearly showing a delayed tumor growth (**Figure 5A**). There were no significant differences in final tumor weight (**Figure 5B**) or spleen weight (**Figure 5C**) in the S100A9 injected mice compared to untreated mice. In contrast to the Paquinimod treatment, S100A9 injection did not cause any clear changes to tumor immune cell infiltration (**Supplementary Figure 7**). Taken together, these findings confirm an early anti-tumor effect of S100A9 in this mouse tumor model.

### 3.6 Paquinimod treatment alters the chemotactic capacity of Ly6C^high^ bone marrow cells *in vitro*

S100A9 is known to induce chemotaxis of neutrophils and MDSCs (9, 31). In the context of COVID-19, the induction of neutrophils by S100A9 was shown to be effectively blocked by Paquinimod (28). In our experiments, early inhibition of S100A9 with Paquinimod caused decrease in overall immune cell infiltration to tumors in CT26 mouse tumor model. Ly6C^high^ monocytic cells in the TME were the population that displayed the strongest reduction, but Ly6G^+^ neutrophils were not affected (**Figure 4B**). To investigate whether Paquinimod affects Ly6C^high^ cell chemotaxis directly, similar to previous work on neutrophils, we performed Boyden chamber assay, testing the migration capacity of Ly6C^high^ monocytes sorted from mouse bone marrow in response to different stimuli with and without Paquinimod. Gating strategy for FACS sorting of Ly6Chigh cells from bone marrow is shown in **Supplementary Figure 8** and results from purity assessment is shown in **Supplementary Figure 9**. We used recombinant S100A9 protein and CT26 tumor cell supernatant (CT26 sup) as migratory stimuli.

**Figure 6:**
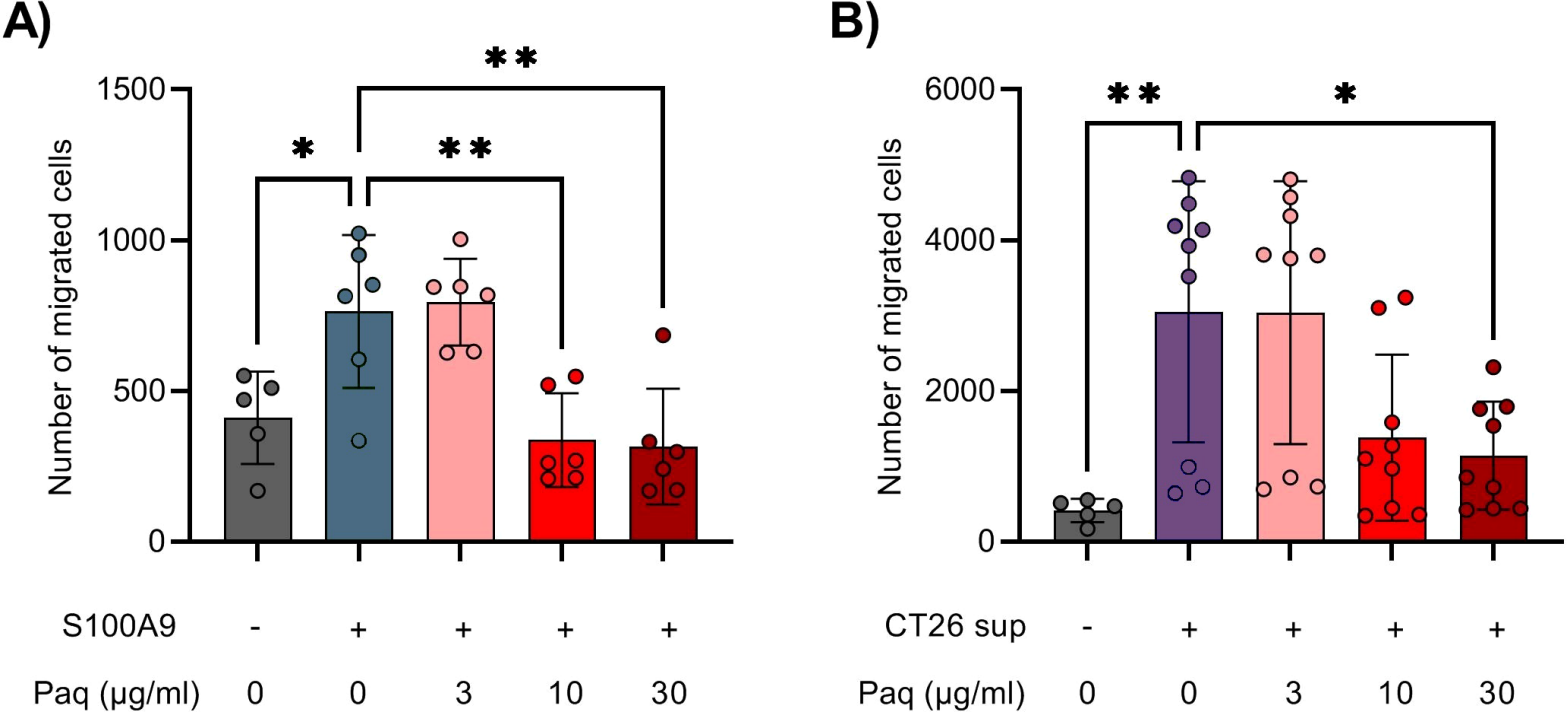
Paquinimod treatment alters the migration capacity of Ly6C^high^ monocytes sorted from bone marrow. **(A, B)** Quantification of migrated Ly6C^high^ bone marrow monocytes into the lower chamber of the Boyden chamber assay. Cells were treated with or without various doses of the inhibitor Paquinimod (Paq). Migration was assessed after 24 hours of incubation at 37°C and 5% CO2. **(A)** Migration response of Ly6C^high^ bone marrow monocytes in response to medium alone (–) or S100A9 (9 µg/ml, +). **(B)** Migration response of Ly6C^high^ bone marrow monocytes in response to medium alone (–) or CT26 tumor cell line supernatant (CT26 sup, +). Figure shows the number of migrated cells stained with NucBlue. Results are expressed as mean ± SD of pooled data from three independent experiments. Statistically significant differences between treatment groups are denoted * or ** depending on p-values as described in methods section.

We found that S100A9 induced statistically significant migration of Ly6C^high^ cells, which was abolished by Paquinimod treatment at either 10 or 30 µg/ml (**Figure 6A**). CT26 supernatant had an even stronger chemotactic effect, which was also significantly inhibited by simultaneous treatment with Paquinimod at 30 µg/ml (**Figure 6B**). Paquinimod treatment *in vitro* did not show any direct cytotoxic effect on Ly6Chigh cells specifically or total immune cells from bone marrow in general (**Supplementary Figures 10, 11**). We conclude that Paquinimod inhibits the chemotactic effect of S100A9 on Ly6Chigh cells, as well as reduce the chemotactic effect of CT26 tumor cell supernatant. These *in vitro* results indicate that Paquinimod likely functions by counteracting the potent migratory signals released from the tumors for the recruitment of proinflammatory Ly6C^high^ monocytes, which would provide a mechanistic explanation for the pro-tumor effect of Paquinimod when administered at earlier stages of tumor development.

## 4 Discussion

In this study, we investigated the feasibility of targeting immune suppressive myeloid cells by blocking S100A9 extracellular signaling using the inhibitor Paquinimod. A role of S100A8, S100A9 or heterodimer S100A8/A9 in the development and function of suppressive MDSCs in cancer was discovered and reported in 2008 by two separate groups (8, 9). Subsequent works have shown that blocking the effect of S100A9, S100A8 or S100A8/A9 in various ways in a tumor setting, can reduce immune suppression and tumor growth and affect the accumulation of myeloid cell populations (**Table 1**). Additional alarmins, such as NLRP-3 or HMGB1, have been shown to mediate resistance mechanisms to checkpoint inhibition or co-activation of antitumor immunity, depending on the study. Running contrary to the apparent anti-inflammatory function in cancer, the S100A8, S100A9 and S100A8/A9 were also reported to mediate chemotactic and pro-inflammatory responses in infection and autoimmunity (6, 36, 37). This conundrum, combined with the controversy regarding the identification of MDSCs as a separate cell type or as a functional phenotype on myeloid cells, warrants further investigation into the role of S100A9 signaling and myeloid immune cells in cancer (38).

**Table 1:** Previously published works into the role of S100A8, S100A9 or heterodimer S100A8/A9 in cancer.

Ref	Blocking strategy	*In vivo* model	Readout and findings
(8)	mAbGB3.1 anti-glycan Ab against RAGE, treatment started after removal of primary tumor	BALB/c, DO11.10, 4T1 mammary carcinoma,	Targeting caused reduced frequency of Gr1+ cells in spleen and lymph nodes, no reduction of T cell suppression per cell. Tumor growth effect not investigated.
(9)	S100A9KO mice and overexpression of S100A8, S100A9 and S100A8/A9 in embryonic stem cells, transgenic mice with overexpression of S100A9.	EL-4 lymphoma, C3 sarcoma, CFA injection	Reduced tumor growth, frequency of myeloid subsets in spleen and differentiation of stem cells into DCs. Reduced differentiation to suppressive myeloid cells vivo and reduced T cell suppression activity.
(21)	Tasquinimod (oral treatment, day 0 or 1 to end of experiment), q-compound targeting TLR4 signaling of S100A9, alone, with SurVaxM peptide and GM-CSF or with tumor-targeted superantigen.	Myc-CaP and B16-h5T4 cell lines,	Reduced tumor growth by combination therapies. Reduced frequency of myeloid subsets in spleen, tumor and blood, and some reduction in T cell suppression by targeting.
(32)	Tasquinimod (oral treatment twice daily) alone or in combination with anti-PD-L1	Bladder cancer: AY-27 in rats, MBT-2 in C3H/HeNRj mice	Reduced tumor growth by single treatment when given early. Effective in TLR4-defective mice. No change frequencies of myeloid or lymphoid immune cells in tumor but change towards an M1/antitumor phenotype. Synergistic effect when combined with CPI.
(33)	Eritoran, and antagonist of TLR4/MD-2 complex. 5 days treatment.	LLC lung carcinoma	Reduced tumor growth and pulmonary recruitment of myeloid cells.
(34)	Neutralizing antibody against S100A8/A9, Ab45	Metastatic melanoma	Treatment reduced lung tropic melanoma metastasis
(35)	S100A8 competitive inhibitory peptide (divalent peptide3A5) against TLR4/MD-2	Syngeneic mouse models	Treatment improved efficacy of anti-PD-1 Ab with reduced lung metastasis

We demonstrate that blocking S100A9 signaling using Paquinimod is detrimental to the initiation of anti-tumor responses in mice models of cancer. This effect was present when treatment was applied early after tumor cells inoculation, and correlated with a strongly reduced infiltration of immune cells into the tumor. The Ly6C^high^ CD11b^+^ myeloid cells, the most numerous immune cell population infiltrating the CT26 tumors at early timepoints, were reduced about 5-fold by Paquinimod. This population might function as suppressive M-MDSCs or proinflammatory monocytes, with the latter being the most probable, based on the increased tumor growth in paquinimod-treated mice. To verify the importance of S100A9 signaling on anti-tumor immunity, we injected recombinant S100A9 into CT26 tumors and did indeed observe a significant anti-tumor effect from day 17 onwards. Finally, we found that Paquinimod reduces the migratory capacity of Ly6C^high^ myeloid cells in response to CT26 tumor cells *in vitro*. We therefore conclude that Paquinimod treatment blocks beneficial proinflammatory and chemotactic signals from S100A9 in cancer.

The pro-tumor effect of Paquinimod was somewhat unexpected, as previous studies targeting S100A8 and/or A9 signaling has demonstrated anti-tumor effects (**Table 1**). The study by Nakhlé et. al, is the most comparable in both targeting and types of readout, as it utilizes Tasquinimod, a compound highly related to Paquinimod, for inhibiting S100A9 signaling (32). However, it differs from our study in regards to the mode of treatment (intraperitoneal injection *vs*. oral in Nakhlé et. al), tumor model (colon carcinoma *vs*. bladder carcinoma) and murine strain (BALB/c *vs.* C3H/HeNRj). Interestingly, the authors find that the anti-tumor effect of Tasquinimod is conserved in TLR4-deficient mice, indicating that Tasquinimod effect might be due to blocking S100A9-signaling through other receptors, or potentially to additional, yet unidentified mechanisms (39). TLR4 was found to mediate the main activation signaling pathway for monocytes by S100A9 (30), although both Tasquinimod and Paquinimod potentially also targets S100A9 signaling through the RAGE receptor, in addition to TLR4 (20). Taken together, investigating the pleiotropic effects of S100A9 is critical, and appears to require multiple targeting strategies and, a clearer understanding of the contribution of various signaling pathways in the initiation and maintenance of anti-tumor responses.

Our study also finds that Paquinimod treatment does not affect the generation or accumulation of suppressive Ly6G^+^ cells, which seems to run contrary to the consensus that S100A9 is important for MDSC development in cancer. However, the conclusions are derived from different experimental settings. For instance, Cheng et al. utilized knockout mouse models to show that S100A9 gene expression was required for development of suppressive MDSCs, and that the lack of S100A9 had a beneficial effect during tumor challenge. S100A9 is a highly multifunctional protein which plays both intracellular and extracellular roles, binds multiple receptors (30) and is regulated by posttranslational modifications, such as oxidation or phosphorylation (3, 40). It is therefore possible that intracellular S100A9 in myeloid cells mediates opposite functions to extracellular S100A9 released by cancer cells or other immune cells, and this possibility remains to be further investigated. Also, Sindha et. al, showed that MDSCs from tumor-bearing mice, synthesize and secrete S100A8/A9, and that *in vivo* treatment using an anti-carboxylated glycan antibody, which was shown to bind S100A8/A9, reduced the accumulation of Gr1^+^CD11b^+^ cells (denoted as MDSCs) in the blood, spleen and lymph nodes. However, such antibody treatment did not abolish the suppressive capacity of the Gr1^+^CD11b^+^ cells. These observations raise the possibility that these cells might very well be a mix of suppressive Ly6G^+^ cells and proinflammatory Ly6C^high^ cells, as the Gr1 antibody recognizes epitopes which are shared between Ly6G and Ly6C. Nevertheless, based on a migration assays, the authors concluded that S100A8/A9 have a chemotactic effect on Gr1^+^CD11b^+^ cells, which is similar to our findings and in agreement with very early finding in the role of S100 proteins in chemotaxis (27, 28).

We observed that Paquinimod treatment and intratumor injection of recombinant S100A9 have opposite anti-tumor effects. This mirror-effect could not be observed only by strictly analyzing the tumor-infiltrating cells, probably as a result of multiple factors. One important point is that Paquinimod treatment was administered daily from day 0 to day 11, while recombinant protein injection could only be performed from day 7 onwards, and was limited to two doses on day 7 and day 11. Generation of a S100A9-overexpressing CT26 tumor cell line or treatment with an oncolytic virus encoding S100A9 could provide continuous release of alarmin at the tumor site and allow investigation of the anti-tumor potential of this signal. Also, we observed a relatively lower impact of S100A9-injection compared with Paquinimod treatment, which might relate to the pleiotropic roles of this protein. It is possible that S100A9 protein injection mediated simultaneous chemotactic/pro-inflammatory effect and immune-regulatory/dampening effects, while Paquinimod mainly changed the balance towards immune-suppression. Despite our attempts to investigate the functional identity of the intratumor Ly6C^high^ myeloid cells by flow cytometry, we still cannot confidently say whether these are proinflammatory monocytes or cells rather similar to the MDSCs. Future studies utilizing proteomics or transcriptomics might enable this distinction, which is an important next step towards tuning the anti-tumor immune response therapeutically. Another caveat is that this study was not designed to elucidate whether the inhibitor Paquinimod worked exclusively through blocking the interaction of S100A9 and TLR4, or whether the effects observed were contributed by interactions with other signaling proteins or receptors. During macrophage activation experiments, we did observe, however, that not all effects of recombinant S100A9 were blocked by Paquinimod. Furthermore, Paquinimod abrogated the migratory capacity of Ly6C^high^ myeloid cells in response to CT26 supernatant, even though these cells released little or no S100A9 (**Figure 6** and data not shown). For both the *in vivo* tumor challenge experiment and the *in vitro* migration assay, we utilized recombinant S100A9 protein and not S100A8 or heterodimeric S100A8/A9. This choice was based on the finding that Paquinimod bound mainly to the S100A9 protein and not S100A8, and the difficulty of using heterodimeric S100A8/A9 *in vitro.* S100A8 and S100A9 spontaneously form tetramers consisting of two S100A8/A9 dimers in calcium-rich conditions such as culture medium or the extracellular space, and these tetramers are unable to signal through TLR4 (41–43). S100A9 homodimers and heterodimers with S100A8 might have different stability and biological function (44), making it important to recognize which proteins were used when comparing studies.

S100A9 expression has been found to correlate with acquired resistance to various cancer therapies, including targeted therapies (45), immunotherapy (46), chemotherapy (47), radiotherapy (48), as well as showing a complex relationship with prognosis, tumor mutational burden (TMB), microsatellite instability (MSI), DNA methylation, and immune cell infiltration across various tumor types (49). These studies highlight the need to study the real-life effect of inhibiting or activating this pathway, as S100A9 emerges as a targetable key signaling molecule in cancer with effects that span all major therapeutic axis. Our study contributes to acknowledging the complex effects of S100A9 on the immune response to cancer, where early signaling appears to contribute to beneficial immune cell infiltration. However, we only utilized syngeneic implanted tumor models, in which tumor cells were injected intradermally and grow rapidly into a local tumor. This allowed us to study the effect of Paquinimod on the initial, early phase of anti-tumor immunity, but this setup is unlikely to accurately represent the slow development and chronic interactions of cancer cells and immune cells in humans. It also does not account for the potential effects of other cancer treatments. It will be critical to find more clinically relevant models, to further investigate the role of S100A9 signaling in anti-tumor immunity and determine how we can best target this pathway to increase accumulation of beneficial immune cells in tumor, while reducing detrimental effects that support tumor progression and therapy resistance.

In this study, we asked if we could reduce suppressive myeloid cells and achieve anti-cancer efficacy by single treatment with Paquinimod or in combination with CPI. Contrary to expectations based on several previous studies, we found that inhibiting S100A9 signaling using Paquinimod reduced beneficial proinflammatory and chemotactic signals in cancer. We therefore conclude that other inhibitors or signaling pathways must be investigated to dismantle the potentially detrimental effects of S100A9 in initiating immune suppression in cancer. Future studies exploring the role of S100A9 as well as other alarmins in cancer is of great importance for the development of new therapeutics aimed at tuning the anti-tumor immune response and for understanding the resistance mechanisms to current treatments such as checkpoint inhibition (50).

## Data Availability

The raw data supporting the conclusions of this article will be made available by the authors, without undue reservation.

## Supplementary material

Supplementary Figure 2 Outline of suppression assay using Ly6G^+^ effector cells from spleen.

Supplementary Figure 3 Gating strategy for identification of immune cell subsets in tumor.

Supplementary Figure 4 Outline of Paquinimod treatment schedule for *in vivo* tumor experiments.

Supplementary Figure 6 Outline of recombinant S100A9 treatment schedule for *in vivo* tumor experiments.

Supplementary Figure 7 Effect of recS100A9 intratumoral injection on tumor-infiltrating immune cells.

Supplementary Figure 8 Flow cytometry staining and gating of bone marrow before and after sorting of Ly6Chigh cells for migration assay.
