# Supplementary material for: Blocking S100A9-signaling is detrimental to the initiation of anti-tumor immunity
Source: Front Immunol. 2024 Oct 21;15:1479502. doi: 10.3389/fimmu.2024.1479502 (PMC11532050; doi:10.3389/fimmu.2024.1479502)
Supplement: Supplementary Figure 2 — Outline of suppression assay using Ly6G+ effector cells from spleen. [file DataSheet1.pdf]

Supplementary Material

1. Supplementary Figures

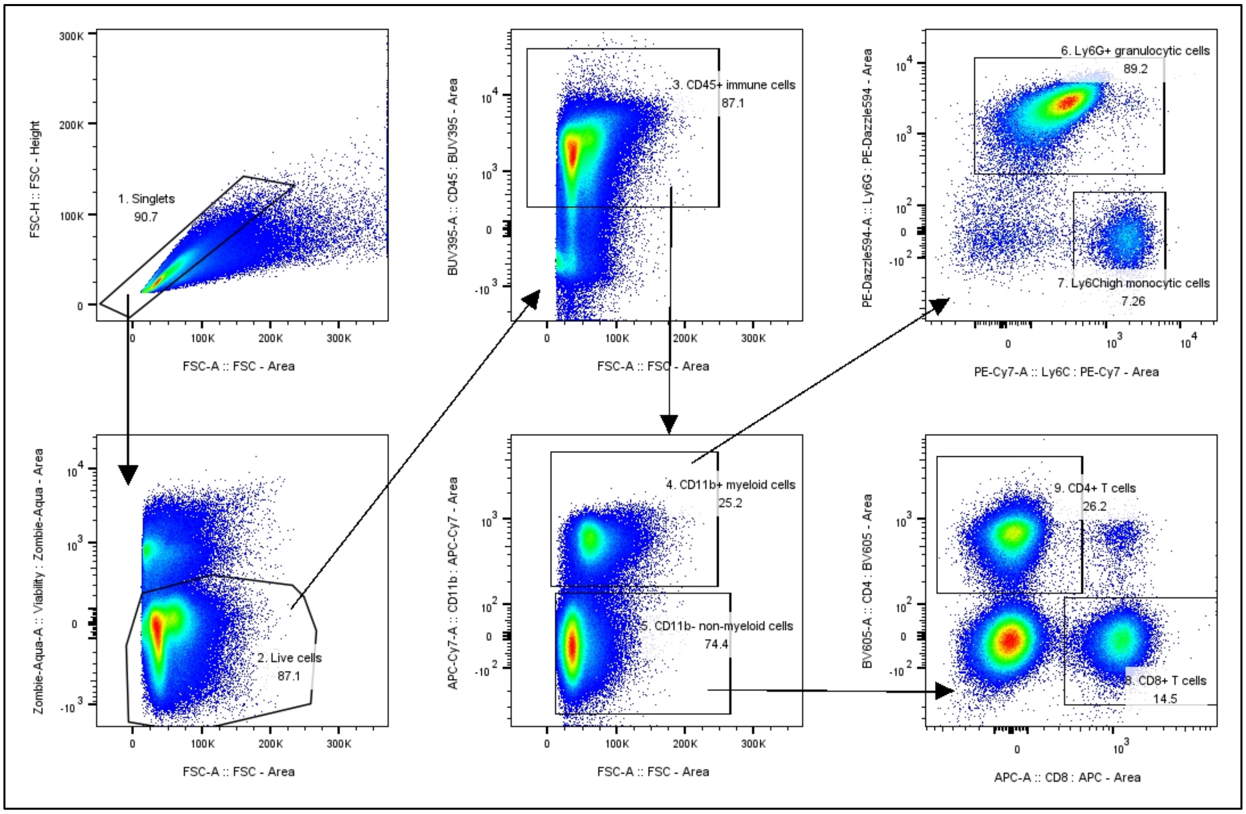

Supplementary Figure 1 - Gating strategy for identification of immune cell subsets in spleen

Figure shows gating strategy identifying the following subsets: 1. Singlets, 2. Live cells, 3. CD45^{+} immune cells, 4. CD11b^{+} myeloid cells, 5. CD11b- non-myeloid cells, 6. Ly6G^{+} granulocytic cells, 7. Ly6C^{high} monocytic cells, 8. CD4^{+} T cells, 9. CD8^{+} T cells

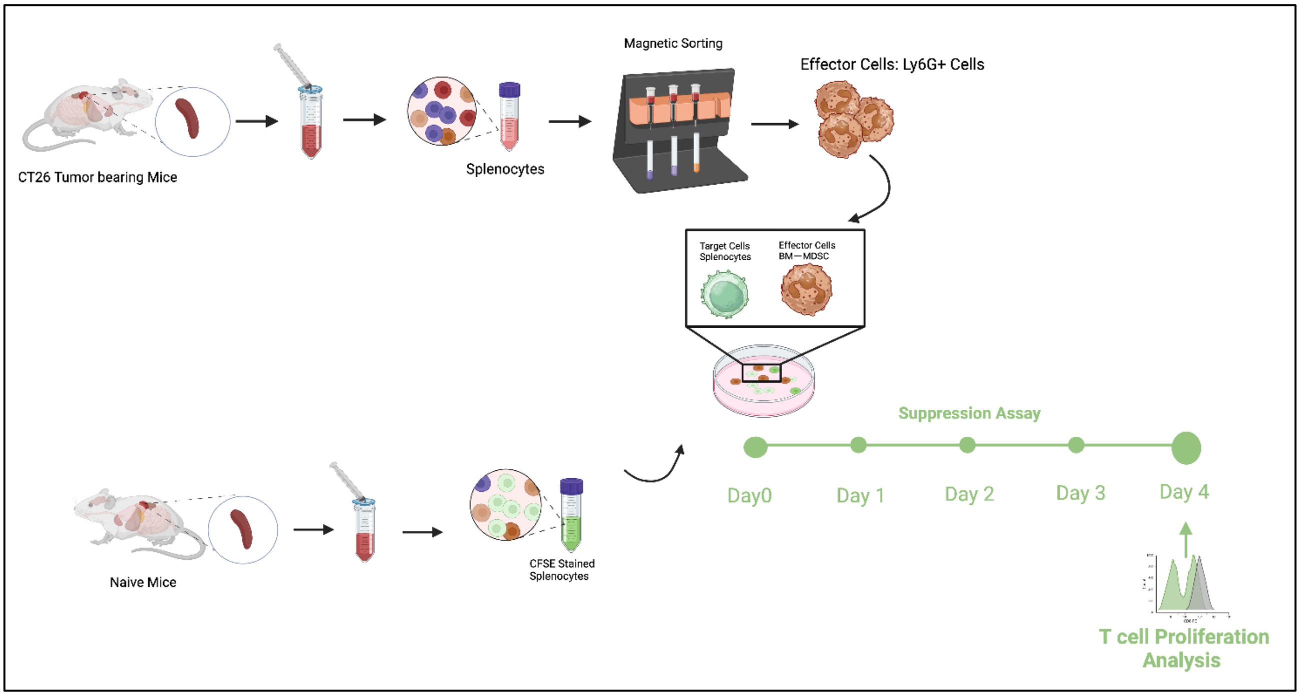

Supplementary Figure 2 – Illustrative Protocol for Isolation of Ly6G+ Effector cells from Spleens and Suppression Assay

Ly6G+ cells from the spleens were isolated from single-cell suspensions using the Anti-Ly-6G MicroBeads UltraPure kit, following the manufacturer's recommended protocols. Spleens from naive, control, and paquinimod treated mice were harvested and pooled based on treatment groups. The spleens were mechanically dissociated by pressing them through a 70 µm cell strainer using a syringe plunger to obtain a single-cell suspension. The cell suspension was centrifuged at 400×g for 5 minutes, and the pellet was resuspended in ACK lysis buffer to lyse red blood cells. After another centrifugation at 400×g for 5 minutes, the cells were resuspended in a buffer containing phosphate-buffered saline (PBS), 0.5% bovine serum albumin (BSA), and 2 mM EDTA. The cells were labeled with Anti-Ly-6G MicroBeads UltraPure by adding 10 µL of MicroBeads per 10^7 cells and incubating at 4°C for 10 minutes. After washing to remove unbound MicroBeads, the cell suspension was applied to a MACS® LS Column placed in the magnetic field of a MACS Separator. The labeled Ly6G+ cells were retained in the column, while unlabeled cells passed through. The column was removed from the magnetic field, and the retained Ly6G+ cells were eluted. Effector cells of interest were seeded in triplicates in varying numbers in U-bottom 96-well plates in RPMI-1640 medium supplemented with 10 % (v/v) heat-inactivated fetal bovine serum (FBS) and 1 % (v/v) penicillin-streptomycin mixed solution. A portion of naïve splenocytes were kept unsorted for use as target cells. Target cells, consisting of total splenocytes harvested from naïve mice, were stained with the CellTrace-CSFE Cell Proliferation kit and added at 200.000 cells/well density to the effector cells, yielding different ratios of effector to target cells. T cells in the splenocyte target population were stimulated by co-culturing together with anti-CD3 and anti-CD28 monoclonal antibody-coated Dynabeads. The co-cultures were incubated for 4 days at 37°C in 5% CO_2. Subsequently, the cells

were harvested, stained for CD11b, CD4 and CD8 and analyzed using the SONY ID7000 spectral cell analyzer. Proliferation was determined by measuring the decrease in CellTrace-CFSE fluorescence intensity in CD4$^+$ and CD8$^+$ T cells. Splenocytes stimulated with anti-CD3 and anti-CD28 monoclonal antibody-coated Dynabeads alone was used as the control and were considered 100% proliferated. Created with BioRender.com

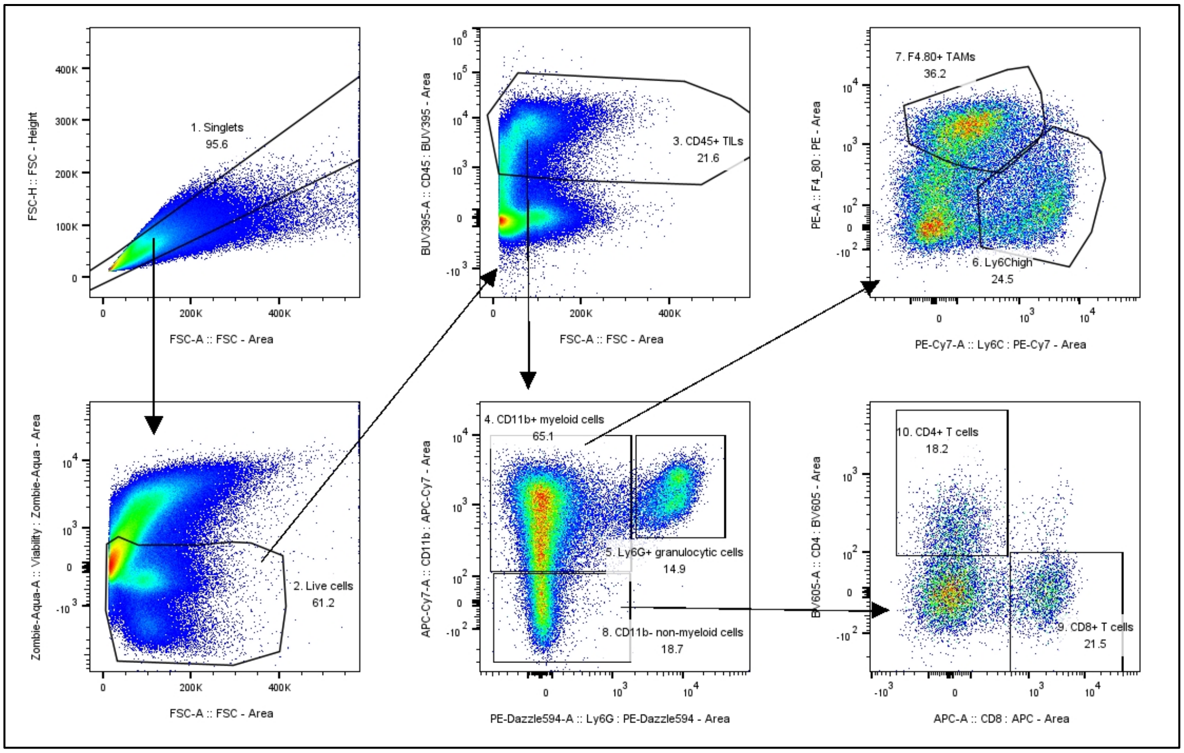

Supplementary Figure 3 – Gating strategy for identification of immune cell subsets in CT26 tumor

The Figure shows gating strategy identifying the following subsets: 1. Singlets, 2. Live cells, 3. CD45$^+$ tumor infiltrating cells (TILs), 4. CD11b$^+$ myeloid cells, 5. Ly6G$^+$ granulocytic cells, 6. Ly6C^{high} monocytic cells, 7. F4/80$^+$ tumor associated macrophages (TAMs), 8. CD11b$^-$ non-myeloid cells, 9. CD8$^+$ T cells, 10. CD4$^+$ T cells

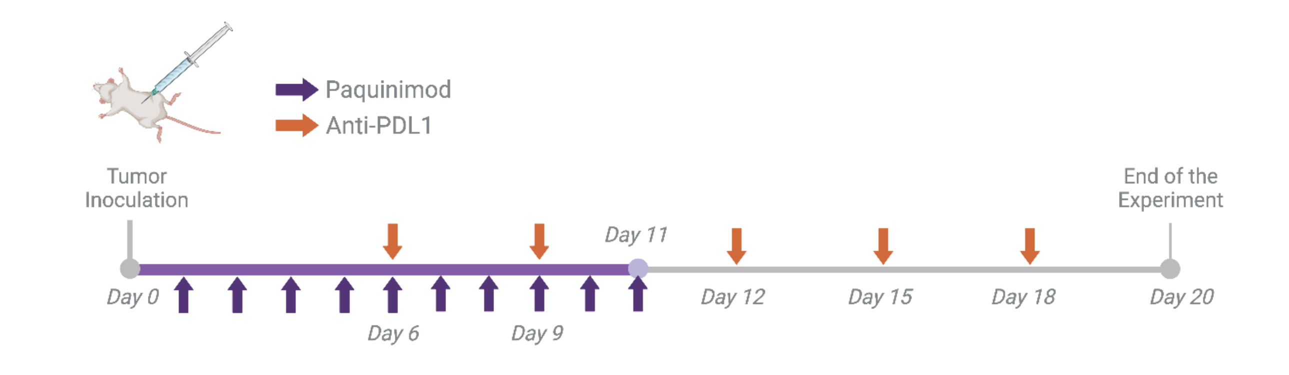

Supplementary Figure 4 – General Outline of in vivo Paquinimod treatment schedule

The figure illustrates the overview of the treatment schedule for *in vivo* tumor experiments with Paquinimod and/or anti-PD-L1. CT26 (1×10^6) cells were injected intradermally (i.d.) into the right flank of BALB/c mice on day 0. Mice were treated with paquinimod (dissolved in DMSO and diluted to 1mg/ml in PBS) by daily intraperitonal (i.p.) injection, from day 0 to day 11. Control mice were injected with solvent (DMSO diluted in PBS). For CPI treatment, mice were injected i.p. with anti-PD-L1 monoclonal antibody (clone 1-111A, produced in-house, 50 µg/mouse) every 3 days starting day 6 after tumor inoculation. Created with BioRender.com

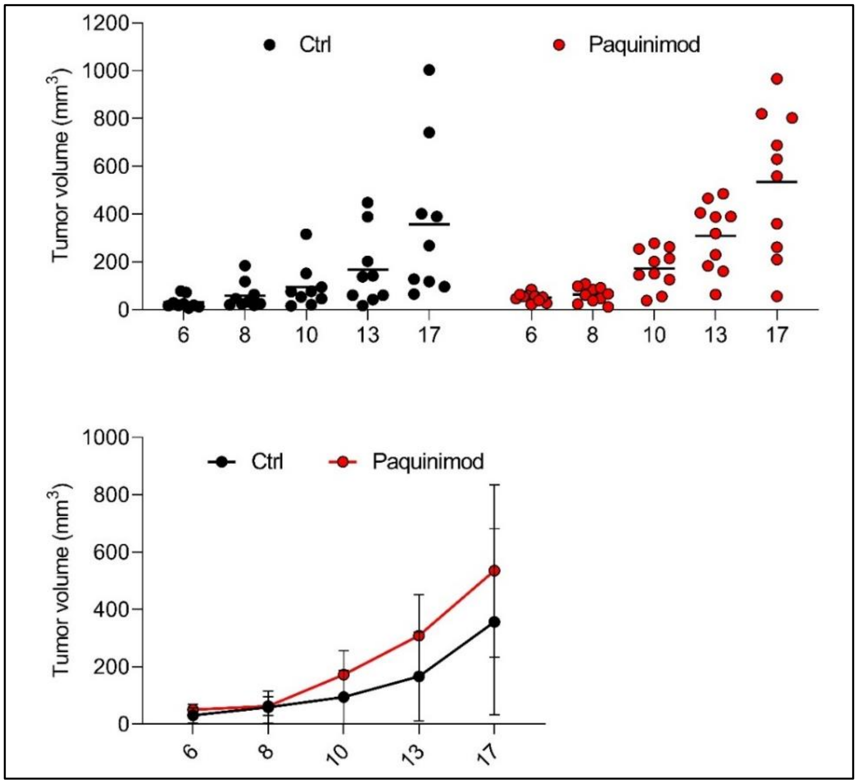

Supplementary Figure 5 – Paquinimod treatment in the LLC model

LLC (5×10^5) cells were injected intradermally (i.d.) into the right flank of C57BL/6N mice on day 0. Mice were treated with Paquinimod day 0-11 or left untreated. Tumors were measured every 2-3 days using electronic calipers, and the tumor volume was calculated using the formula: tumor volume $= \pi \times (\text{length} \times \text{breadth} \times \text{height})/6$. N=9 and 10. Graph shows representative experiment from 3 repeats. Upper graph shows growth of LLC tumors in individual mice, grouped depending on treatment and response. Lower graph shows growth of LLC tumors as average $^+/-$ st.d.

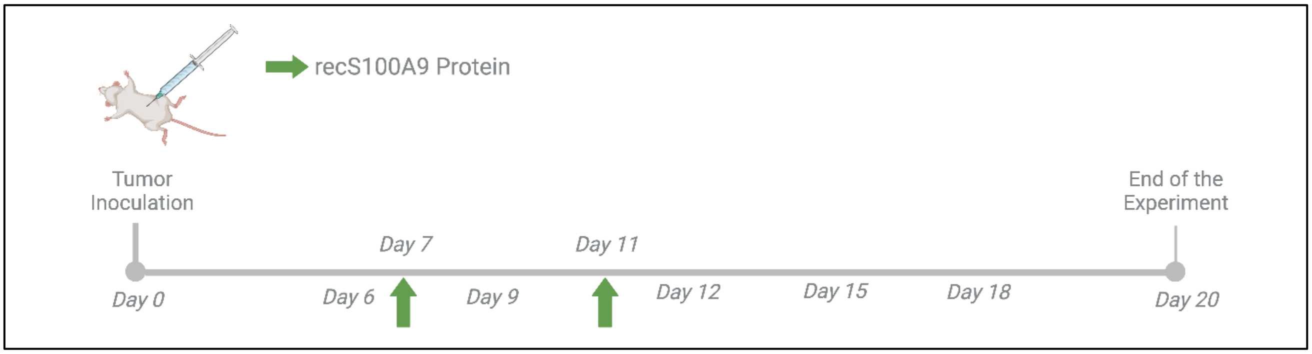

Supplementary Figure 6 – General Outline of in vivo recombinant S100A9 treatment schedule

The figure illustrates an overview of the treatment schedule of CT26 tumor bearing mice with recombinant mouse S100A9 protein. CT26 (1×10^6) cells were injected intradermally (i.d.) into the right flank of BALB/c mice. Recombinant mouse S100A9 protein (2 µg/mouse in 20 µl volume) was administered intratumorally on days 7 and 11. Control mice received an intratumoral injection of an equal volume (20 µL) of PBS. Tumor volumes were measured every other day using electronic calipers, and the tumor volume was calculated using the formula: tumor volume = $\pi \times$ (length $\times$ breadth $\times$ height)/6. Mice were sacrificed on day 19-21 for growth curve analysis. Created with BioRender.com

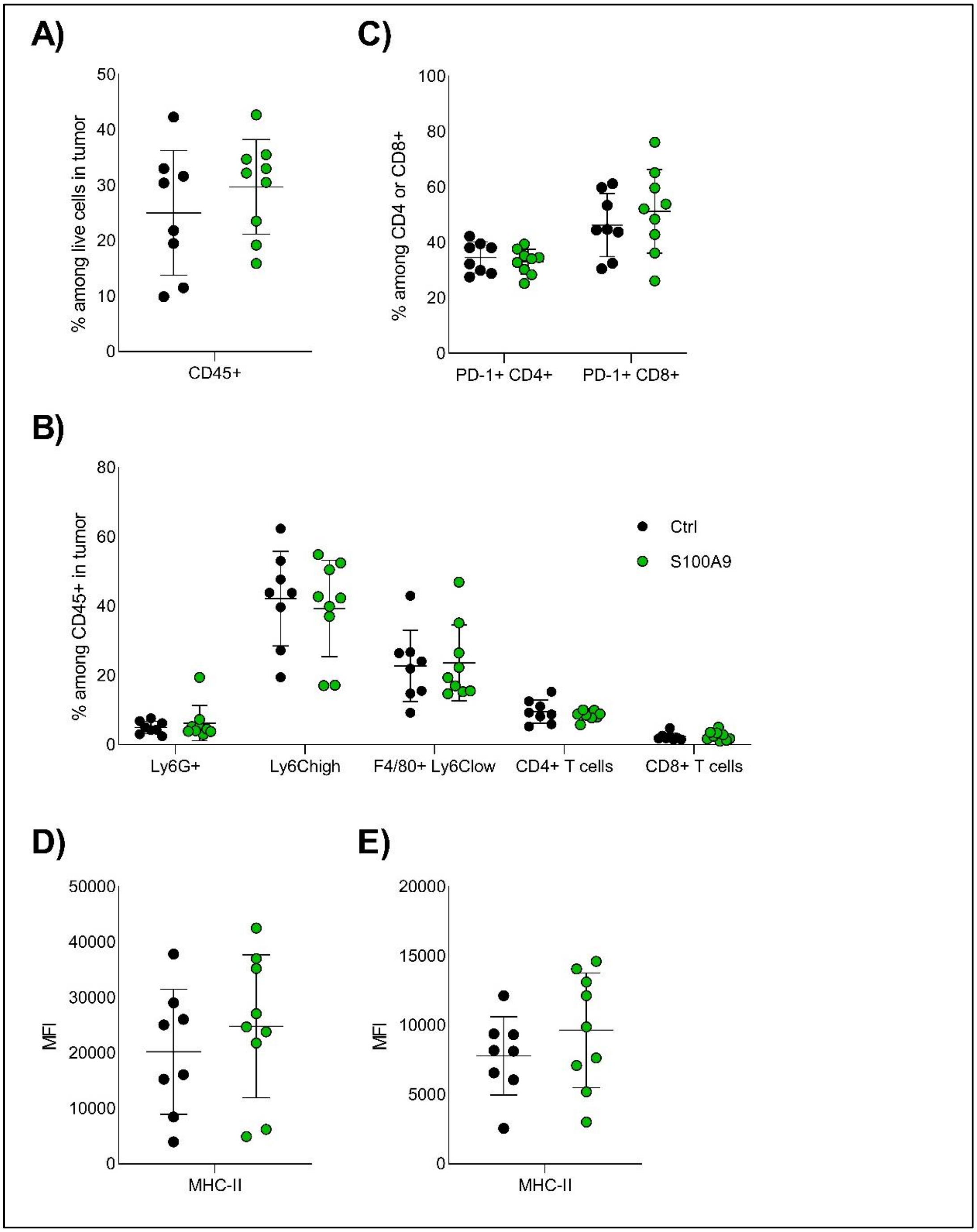

Supplementary Figure 7 – Effect of Recombinant S100A9 Protein Treatment on Immune Cell Populations in CT26 Tumors

Figure shows flow cytometry analysis CT26 tumors of mice treated with recombinant mouse S100A9 at day 12. CT26 (1×10^6) cells were injected intradermally (i.d.) into the right flank of BALB/c mice. Recombinant mouse S100A9 protein (2 µg/mouse in 20 µl volume) was administered intratumorally on days 7 and 11. Control mice received an intratumoral injection of an equal volume (20 µL) of PBS. Mice were sacrificed on day 12 for flow cytometry analysis. N=8 mice for untreated control

and 9 for recS100A9. Graph shows representative experiment from 3 repeats. **A)** Frequency of total immune cells in tumors, identified as CD45$^+$ among live cells by flow cytometry. **B)** Frequency of immune cell subsets among total CD45$^+$ cells in tumors. **C)** Frequency of T cell subsets among total CD45$^+$ cells in tumors. **D)** Mean fluorescence intensity (MFI) of MHC-II on TAMs from tumors. **E)** Mean fluorescence intensity (MFI) of MHC-II on Ly6C^{high} cells from tumors.

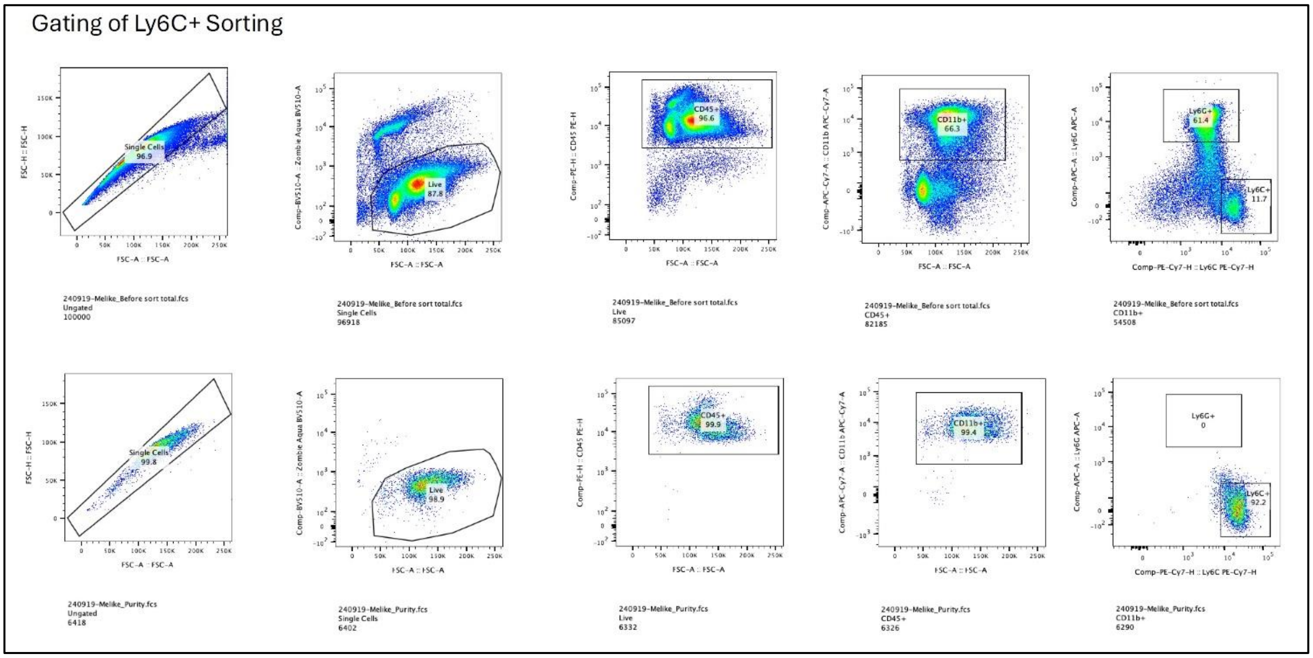

Supplementary Figure 8 – Flow cytometry staining and gating of bone marrow before and after sorting of Ly6C^{high} cells for migration assay

Upper panel shows single cells suspension from bone marrow before sort, identifying the target population of Ly6C+ CD11b+ CD45+ live cells. Lower panel shows the purity analysis with flow cytometry staining of cells after sorting.

	Frequency of Ly6C+ among total cells
Before sorting	6.38%
After sorting	90.4%

Supplementary Figure 9 – Final purity assessment of Ly6C+ sort

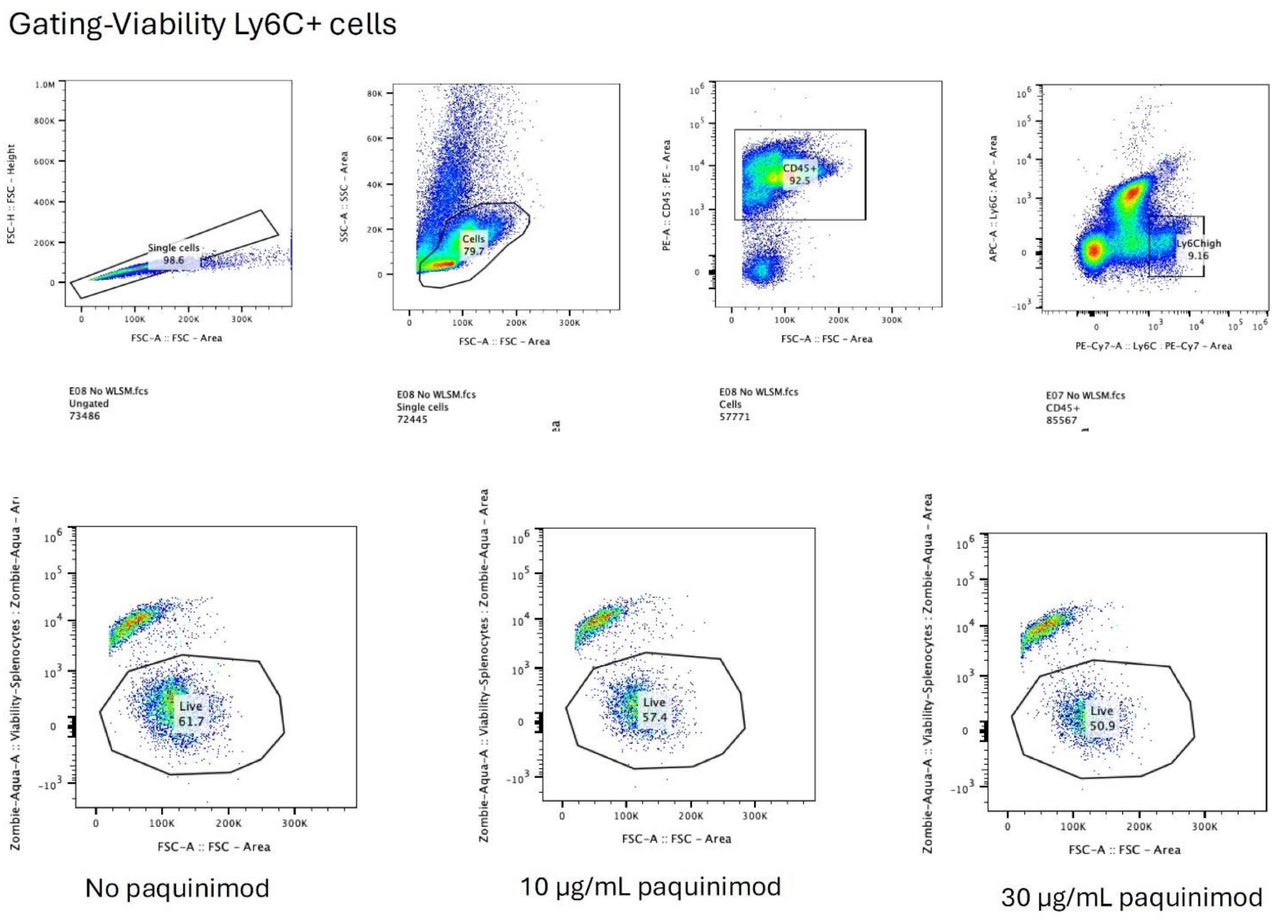

Percentage of Live Cells (ZombieAqua negative) Freq of Ly6C high	Average
Control	61.95
10 µg/mL paquinimod	57.15
30 µg/mL paquinimod	50.45

	Relative to Control %
Control	100
Paquinimod 10 µg/mL	92.251816
Paquinimod 30 µg/mL	88.2764654

Supplementary Figure 10 – Viability analysis of Ly6C+ cells after *in vitro* incubation with paquinimod

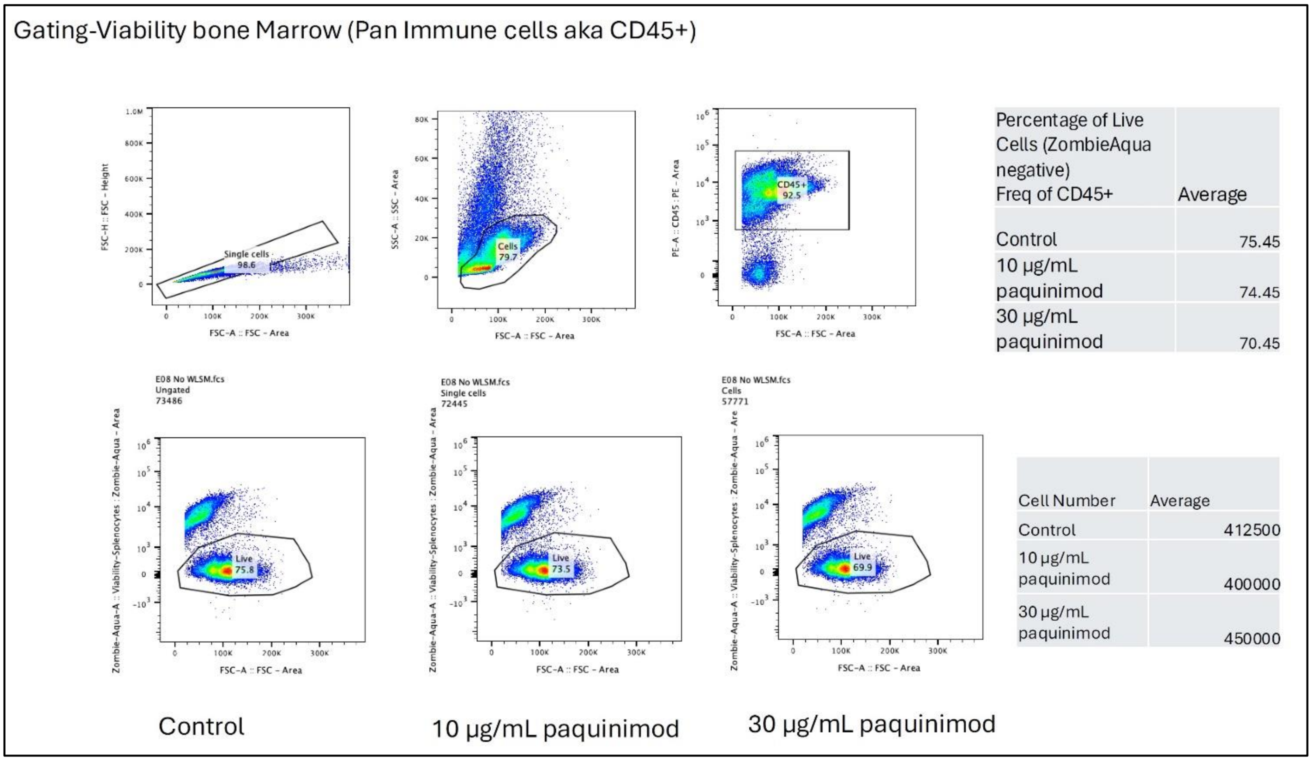

Supplementary Figure 11 – Viability analysis of total bone marrow immune cells after *in vitro* incubation with paquinimod
